# Modern Approaches in Organic Chromofluorescent Sensor Synthesis for the Detection of Considered First-Row Transition Metal Ions

**DOI:** 10.3390/molecules30061263

**Published:** 2025-03-12

**Authors:** Samina Aslam, Iram Kousar, Sadia Rani, Isra Zainab, Sadia Bristy, Rachid Skouta

**Affiliations:** 1 Department of Chemistry, The Women University Multan, Multan 66000, Pakistan; kousariram361@gmail.com (I.K.); sadiarani112wb@gmail.com (S.R.); israzainab55@gmail.com (I.Z.); 2 Department of Chemistry, University of Massachusetts, Amherst, MA 01003, USA; sbristy@umass.edu; 3 Department of Biology, University of Massachusetts, Amherst, MA 01003, USA

**Keywords:** chemical sensors, environmental monitoring, biological detection, sensor design

## Abstract

The development of optical chemosensors for the sensitive and selective detection of trace-level metal ions in aqueous solutions has drawn a lot of attention from the scientific community in recent years. Organic sensors offer a number of advantages over traditional identification techniques, including low cost, high sensitivity, selectivity, and simplicity of synthesis. We will focus on colorimetric and fluorometric sensors based on their receptors for the real-time detection of certain first-row transition metal ions like Cr^3+^, Mn^2+^, Fe^2+^, Co^2+^, Ni^2+^ Cu^2+^, and Zn^2+^. The development of these sensors will aid in the rapid and simple resolution of several problems linked to the detection of potentially hazardous metal ions at trace levels in diverse biological and environmental components. This review article not only gives a comprehensive understanding of the existing techniques, but also encourages more research efforts to address the evolving demands in the field of trace transition metal ion detection.

## 1. Introduction

**Figure 1:**
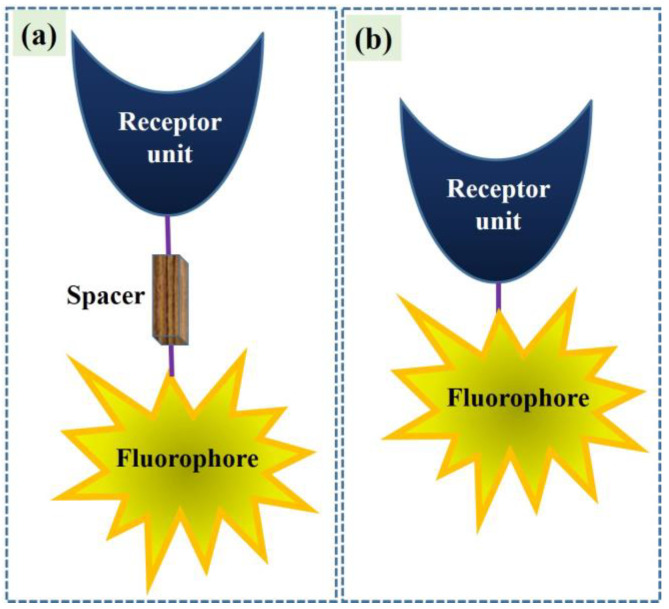
Designing of fluorescence signaling unit (**a**) via spacer signaling unit and via (**b**) integrated signaling unit. Reproduced from ref. [31].

Modern research communities are particularly interested in building single organic molecules that can detect a range of metal ions using a number of analytical techniques, as a result of the current surge in chemosensor innovation [1,2,3]. These sensing compounds might become crucial due to their important role in biological systems and very dangerous impact on the environment [4,5]. The development of sensors for electrochemical stripping analysis of heavy metals is an emerging research field due to the increasing need for simple-to-use and cost-effective devices for environmental monitoring and other industrial applications. Many works concerning heavy metal sensing based on the use of carbon nanostructured materials, such as carbon nanotubes(CNTs), have been reported so far. In addition, modifications using nanoparticles, such as cadmium, palladium, lead, bismuth nanoparticles and goldnanoparticles, or other nanostructured materials, have also been reported for their use in heavy metal electrochemical detection [6,7,8,9] A chemosensor is a molecule that combines with an analyte to cause a measurable change. A chemosensor is made up of a receptor and a detector. The signal of the detector changes when the receptor connects with the metal. Fluorescent chemosensors are an important category of chemosensors that monitor hazardous heavy and transition metals in the environment by using fluorescence as the output signal. Over the past few decades, a large number of research publications and review articles have been focused on fluorescent chemosensors for the detection of toxic heavy and transition metals [10]. Various traditional analytical techniques require time-consuming processes, costly equipment, and intricate sample preparation [11]. In contrast to conventional analytical techniques (e.g., spectrophotometric approach [12], colorimetric detection [13], atomic absorption spectrometry [14], flow injection spectrophotometry [15], reversed-phase high performance liquid chromatography [16], electrochemical probe [17]), the approaches based on fluorescent chemosensors have garnered special interest because of their great sensitivity and selectivity, as well as their ease of use and simplicity [18]. The design and synthesis of sensors with excellent selectivity, sensitivity, low detection limit, and immediate response have required a lot of work [19,20]. In recent years, low-cost chemical sensors have become essential for tracking a variety of species that are significant to the environment and human health, including first-row transition metal ions [21]. Due to their sensitive and selective characteristics, fluorescence sensors are regarded as one of the practical instruments among the many sensors for detecting metal ions [22]. Moreover, fluorescent chemosensors could be applied to living organisms for bio-imaging [23]. Multiple analyte monitoring must be performed concurrently and quantitatively since biological and environmental samples typically comprise a variety of chemicals or ions. It appears to be solvable by loading many probes at once. However, combining many probes might result in photobleaching, a bigger invasive impact, and cross-talk, making the situation complex and inappropriate for quantitative study [24]. The challenges of loading several indicators can be solved if a single probe is able to detect multiple analytes with distinct spectral responses. Therefore, even though the fluorescent probe was created for individual analytes, it is imperative to concurrently detect several chemical analytes in both in vivo and environmental systems [25]. In reality, certain single-molecular multi-analyte sensors are quite good, and some of them can detect two metal ions in a single channel at the same time in aqueous circumstances [26]. Transition metal ions are essential to human existence and directly affect cell activity, especially trivalent metal ions like Cr^3+^ or Fe^3+^ [27]. Many transition metal ions are found in nature and serve a variety of biological purposes. An excessive buildup of these ions in biosystems can cause illnesses and even death in the worst-case scenario [28,29]. Therefore, it is crucial to understand their temporal and geographical distribution both inside and outside of cells in order to comprehend physiological situations. A sensitive method for accurately detecting and measuring an analyte’s concentration is fluorescence spectroscopy [30]. However, the fundamental structure of the signaling system is made up of a guest-binding (receptor) and signaling (fluorophore) moieties that can be covalently or integrated through a spacer (Figure 1).

The fluorescence signaling unit with a spacer (Figure 1a) is better than the integrated unit (Figure 1b) because it allows one to change the fluorophore, the receptor, or both at once. A chemosensor that can identify a specific metal ion in the presence of several competing ones is perfect. It should be created using either novel recognition principles or improved ones, which are both extremely difficult tasks for chemists. It is necessary to strategically position suitable donor atoms to match the preferred coordination of the metal ions. Emission is efficiently quenched by biologically relevant paramagnetic transition metal ions, and this signaling mechanism has frequently been used to identify the presence of an analyte [32]. For practical and sensitivity reasons, it is preferable to choose a method that increases fluorescence rather than quenching when designing a sensor. In other words, to prevent the quenching of fluorescence, a paramagnetic transition metal ion needs to have a stronger metal ion-receptor (M–R) connection than metal ion–fluorophore (M–F) interaction. The general architecture of the signaling system may be designed with this concept in mind. Despite significant advancements in this area, new techniques for metal ion detection are still required. Compared to conventional identification methods, organic sensors have several benefits, such as cheap cost, high sensitivity, selectivity, and ease of synthesis [33].

### Colorimetric and Fluorometric Detections

In the domain of chemosensors, colorimetric detection is the simplest technique. Using a basic visible spectrophotometer, the colorimetric enabled investigation that can be seen with the naked eye [34]. In the colorimetric approach, a chromophore group serving as the signaling unit in a chemosensor exhibited a distinct color scheme. The chromophore displays its characteristic color in its unbound state as well as the precise color shift that occurs during analyte interaction. Thus, after the analyte is added, the colorimetric method measures the change in absorbance spectrum [35]. The colorimetric approach therefore has limitations such as that it cannot detect background interference or low sensitivity. The fluorometric method is better fitted for practical use because of its higher sensitivity than that of the colorimetric method. A common technique for identifying and measuring cations, anions, or neutral substances is the fluorometric strategic approach. In chemosensor applications, the fluorometric approach employs a chemical moiety with a fluorophore unit [36,37].

**Figure 2:**
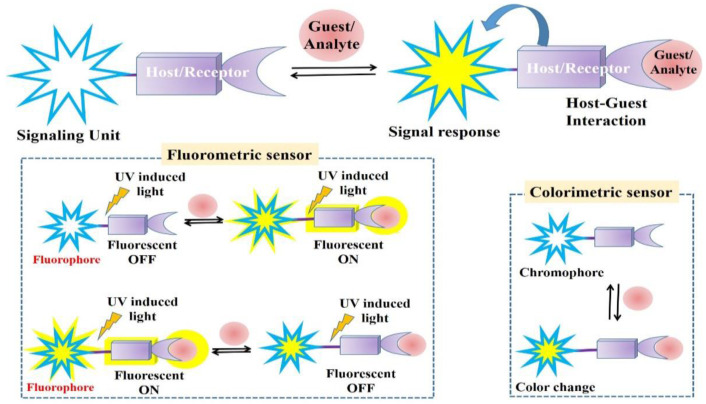
The overall detection mechanism of colorimetric and fluorometric chemosensor agents. Reproduced from ref. [37] with permission from Pandawa Institute, copyright 2022.

Colorimetric chemosensors are used within absorbance spectroscopy to detect a visual color change caused by analyte/guest binding. On the other hand, a fluorometric chemosensor is an analyte/guest that can interact via a variety of sensing methods [38]. Figure 2 shows schematic illustrations for all general sensing mechanisms. The defined phenomena result in distinct pathways for the detection of particular analytes. However, there are two main categories of detection utilizing the fluorometric method: (1) fluorescence “ON” and (2) fluorescence “OFF”. When a specific analyte was added, the free chemosensor agent’s emission intensity was quenched, causing the fluorescence to go “OFF”. In the meantime, fluorescence “ON” increases the emission intensity when the chemosensor agent and analyte interact.

**Figure 3:**
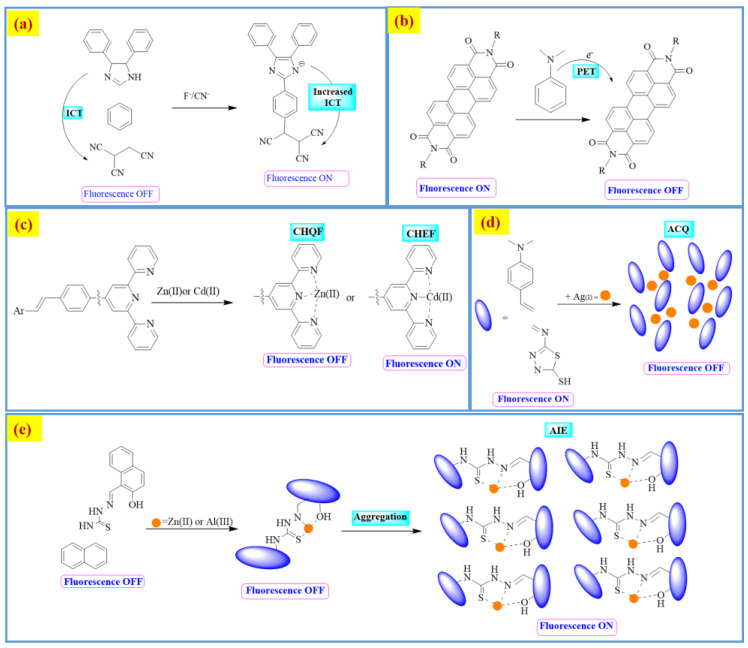
Schematic illustrations of general fluorometric chemosensing mechanisms: (**a**) Intramolecular charge transfer (ICT), where electron movement between donor and acceptor groups alters the fluorescence emission. (**b**) Photo-induced electron transfer (PET), where electron transfer between the fluorophore and analyte results in fluorescence quenching or enhancement. (**c**) Chelation-enhanced fluorescence (CHEF) and chelation-quenched fluorescence (CHQF), illustrating the effect of metal ion coordination on the fluorescence intensity. (**d**) Aggregation-caused quenching (ACQ), where the fluorescence intensity decreases due to the formation of aggregates. (**e**) Aggregation-induced emission (AIE), where aggregation leads to enhanced fluorescence due to restricted intramolecular motion. Copied from ref. [37] with permission from Pandawa Institute, copyright 2022.

Figure 3 presents schematic illustrations of general fluorometric chemosensing mechanisms, including intramolecular charge transfer (ICT) (Figure 3a) [38], photo-induced electron transfer (PET) (Figure 3b), chelation-enhanced fluorescence (CHEF), chelation-quenched fluorescence (CHQF) (Figure 3c), aggregation-caused quenching (ACQ) (Figure 3d) [39], and aggregation-induced emission (AIE) (Figure 3e) [40]. These defined phenomena lead to distinct pathways for detecting specific analytes [41]. The simplicity and low detection limits that can be attained by employing this technique make sensors based on metal ion-induced changes in fluorescence particularly appealing and the first choice. Therefore, the creation of novel compounds that selectively bind metal ions by fluorescence signal transduction has attracted interest from all around the world. The high ring density of π electrons and the rigid conjugated structure of aromatic compounds are the main causes of fluorescence. Metal ion complexation can lead to either decreased fluorescence-chelation-enhanced quenching (CHEQ) or increased fluorescence, commonly known as chelation-enhanced fluorescence (CHEF). Charge transfer (CT) and electron transfer (ET) are two main mechanisms used to explain how fluorescence sensors react to heavy metals. Photoinduced electron transport (PET), intramolecular charge transfer (ICT), and photoinduced charge transfer (PCT) are examples of these two broad groups. For the detection of heavy metal ions, chemiluminescence and electrochemiluminescence are also used in addition to fluorescence approaches [42].

Various strategies are being employed in order to design the desired binding moiety in a chemosensor for the recognition and detection of the targeted analyte. Organic heterocyclic compounds are helpful in the construction of chemosensors due to their excellent photophysical properties, very low detection limit, high selectivity, synthetic flexibility, reversibility, biocompatibility, and utilization in a wide pH range. These compounds, which have donor atoms like N, O, and S, are highly significant because of their capability of interacting with various metal ions and producing detectable analytical signals [43]. Scientists have been working to build chemosensors using various organic compounds because of their critical need [44]. Among these is the class of colorimetric sensors, which allow metal ion detection with inexpensive equipment [45,46]. Colorimetric sensors that include imine and azide groups are easily synthesized and have a strong binding capacity to metal ions. When aryl-substituted molecukes are bound together, a conjugated system can form and their absorbance is close to or in the visible light spectrum range [47,48]. A common binding group for metal ions chemosensors is the amide group, which also includes thioamide [49]. Protons in thioamide bonds are more potent hydrogen bond donors than those in oxo amide bonds. Compared to an oxo amide, the (C=S)NH proton has a far higher acidity [50]. Taking into account that the thiourea group exhibited an ideal binding moiety and signaling unit [51], several attempts have already been made to design fluorescence sensors that use thiol deprotonation to detect metal ions [52], cyclization [53], and ring-opening [54] in thiourea derivatives. However, each of these chemosensors has a few limitations, such as multiple ion selectivity or a trace level inability to detect in an aqueous media. Thus, there is ongoing interest in the development of fluorescent probes that can selectively remove a certain metal ion without affecting other metal ions [55,56]. Using thiocarbohydrazide as a fluorescent chemosensor, a straightforward Schiff base may selectively identify ions in a semi-aqueous media [57,58]. Chemosensors containing hydrazone–hydrazide moieties are appealing choices due to their high sensitivity and selectivity in detecting a variety of cations and anions [59,60]. Fluorescent sensors have drawn a lot of interest from researchers in the last several years due to their various benefits including nondestructive sample examination, cheap instrument cost, high sensitivity, and outstanding selectivity. Fluorescent sensors typically track their signal as a shift in emission wavelength, fluorescence lifespan, or emission strength in response to minute environmental changes [61]. On the other hand, the development of highly selective ratio metric and “turn-on” fluorescence probes is more important since they can identify metal ions even in living systems, which have been demonstrated to display unique emission characteristics upon metal binding [62]. Schiff base compounds are a novel subclass of chemosensors. These are made up of compounds with a carbonyl group (C=O) and a hydrazone group (N=NCH). Additionally, Schiff bases are crucial to binding chemistry because of their great affinity for metal ions, which are commonly utilized in recognition. This makes them excellent chemosensors. Schiff-based fluorescence chemosensors have received a lot of interest due to their ease of synthesis, cost-effective raw materials, and varied architectures [63]. For many years, the design of novel chemosensors has been of great interest because of their diverse medical aptitude, which includes antibacterial, antiviral, antifungal, anticancer, and anti-inflammatory activity [64].

## 2. Detection of Selected First-Row Transition Metal Ions

**Scheme 1:**
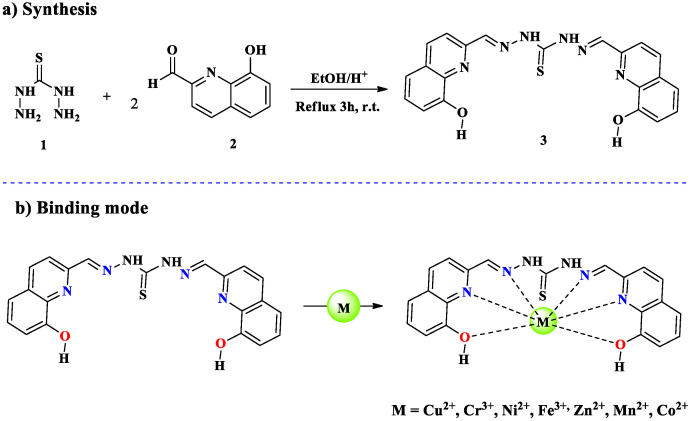
(**a**) Synthesis of Aryl-substituted Thiocarbohydrazide based sensor **3**, (**b**) proposed binding mode with various metal ions.

In analytical chemistry, thiocarbohydrazide is utilized for the identification and measurement of inorganic and organic substances. Thiocarbohydrazide may effortlessly be produced in a laboratory by reacting carbon disulfide with hydrazine hydrate [65,66]. Thiocarbohydrazide effectively precipitates aldehydes and ketones quantitatively, generating a large number of derivatives with high melting points that are utilized in gravimetric procedures and for identification or detection. Thermobalance was employed to determine their thermal stability, and their ideal drying temperatures have thus been identified. It was examined that combining carbohydrazide, pyarazole and naphthaldehyde moitieties through suitable linkage would result in intriguing multifunctional sensing compounds for the optical sensing of various metal ions and anions because of the structural properties of these valuable moieties that can be modified to generate effective binding sites [67,68,69]. Several color tests for anions and metal ions rely on the complexing ability of these chemical compounds. More recent reports have included the interaction of carbohydrazide with Cu and of thiocarbohydrazide with first-row transition metal ions such as Cr^3+^, Mn^2+^, Fe^2+^, Co^2+^, Ni^2+^ Cu^2+^, and Zn^2+^. The study of metal complexes of various thiocarbohydrazide derivatives has been used to examine the binding sites and their molecular structure. Thus, it is evident that thiocarbohydrazide chelates two nitrogen atoms to form complexes with metal ions such as Cr^3+^, Mn^2+^, Fe^2+^, Co^2+^, Ni^2+^, Cu^2+^ and Zn^2+^ in an acidic medium solution [70]. This mini-review represents a broad overview of organic compounds functioning in colorimetric and fluorescent chemosensors for the identification and detection of first-row transition metal ions. The main focus is on the synthesis of Aryl-substituted organic-based chemosensors, which have shown great promise in detecting a wide range of important first-row transition metal ions including zinc, copper, cobalt, chromium, nickel, manganese, and iron through colorimetric and fluorometric turn-off and turn-on responses. Thiocarbohydrazide **1** was used to add the amine and aldehydes in the presence of ethanol in a schiff-based reaction. At room temperature, the reaction mixture was stirred for three hours following the gradual addition of 8-hydroxy quinoline-2-carbaldehyde **2** and a catalytic amount of acetic acid. The precipitates removed the original ingredients in the reaction mass after they were obtained. Precipitates were collected by filtering, EtOH washing, drying in a hot air oven, cooling, recording, and computation of the percentage yield of synthesized chemosensors (Scheme 1). Using Bene-si-Hildebrand (B-H) plots, and UV–visible spectroscopy titration studies, the detection limit (DL), stoichiometric binding ratio, and recognition process were investigated. Sensor 3 demonstrated a 1:1 binding ratio with an excellent binding constant range of 103 to 105 M^−1^ with Cu^2+^ metal ions [71].

**Scheme 2:**
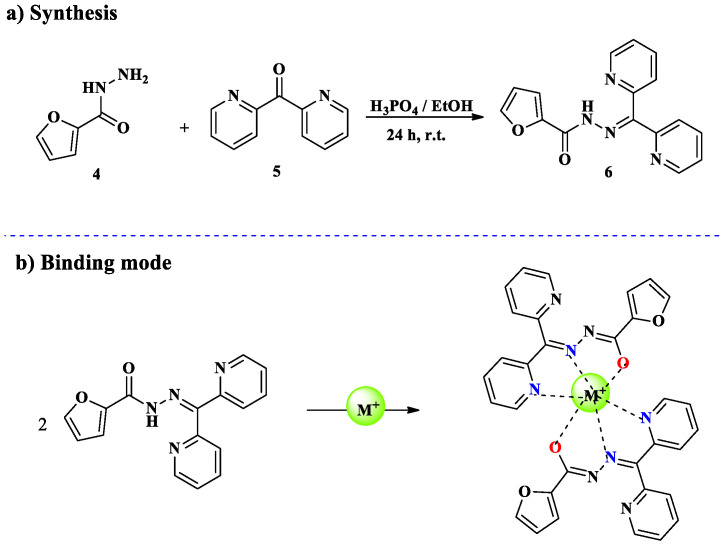
(**a**) Synthesis of carbohydrazide-based sensor **6**, (**b**) proposed binding mode.

M. Yang, J. B. Chae and their coworkers synthesized a novel sensor by reacting 2-furoic hydrazide **4** and di-2-pyridylketone **5** in EtOH. The synthesis procedure involved first dissolving 2-furoic hydrazide in EtOH, and then mixing a few drops of phosphoric acid and di-2-pyridyl ketone into the ethanol solution. After agitating the mixture for a full day at room temperature, a white precipitate formed that was filtered and washed with ether to yield 44% of the desired solid product **6** (Scheme 2). This sensor changed from colorless to blue in the aqueous solution, demonstrating exceptional selectivity and sensitivity for detecting lower concentrations of Fe cations. In addition, the strong binding capability of the sensor in detecting Fe(II), Co(II), and Cu(II) selectively makes it suitable to detect and quantify small quantities of said metal ions in tap water. Sensor **6** shows no visible response to other metal ions such Ca^2+^, Cr^3+^, Mn^2+^, Fe^3+^, Ni^2+^, Zn^2+^, Cd^2+^, Ag^+^, Hg^2+^, and Pb^2+^. The binding ratio of sensor **6** to Fe^2+^, Co^2+^, and Cu^2+^ is 2(sensors):1 (metal ion). The binding constants of the sensor are as follows: Fe^2+^: 1.0 × 109 M^−2^, Co^2+^: 2 × 10^9^ M^−2^, and Cu^2+^: 3.0 × 10^9^ M^−2^. Sensor **6** works well at a neutral pH, and micromolar concentrations of Fe^2+^, Co^2+^, and Cu^2+^ can be detected in the water samples. The sensor’s color response to Cu^2+^ is uniquely attenuated by glutathione [47]. In the case of the sensor, the main absorption band originated from the HOMO → LUMO transition, indicating a π → π* transition. In contrast, the Cu–sensor complex showed an intramolecular charge transfer (ICT) [72].

**Scheme 3:**
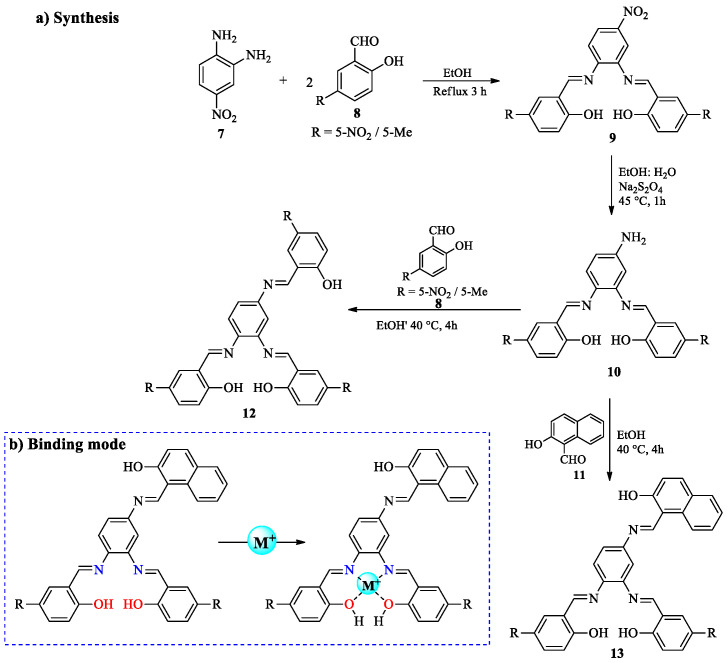
(**a**) Synthesis of Schiff-based chemosensor **13**, (**b**) proposed binding mode.

Özlem Özdemir successfully synthesized a novel chemosensor for the identification and detection of metal ions. She designed diimine-Schiff-based **9** by condensing 4-nitro-o-phenylenediamine **7** with 5-chloro or 5-methyl substituted salicylaldehyde **8**. Then, nitro-derivative **9** was converted into the amino derivative **10** by adding sodium dithionite. Subsequently, she further condensed **10** with **8** and 2-hydroxy-1-nephthaldehyde **11** to give **12** and **13**, respectively. These recently designed Schiff bases, which include ONNO or O_3_N_3_ donor atoms, are resistant and soluble in various organic solvents, like DMSO, DMF, and alcohols at low concentrations (Scheme 3). The properties of all Schiff bases under sensor **13** were examined upon addition of the metal ions, such as Cr^3+^, Fe^2+^, Fe^3+^, Co^2+^, Ni^2+^, Cu^2+^, Zn^2+^, and Pb^2+^. The interactions between receptors and ions are easily monitored via UV–Vis spectroscopy [73]. 

### 2.1. Cr^3+^ Ion Detection

**Scheme 4:**
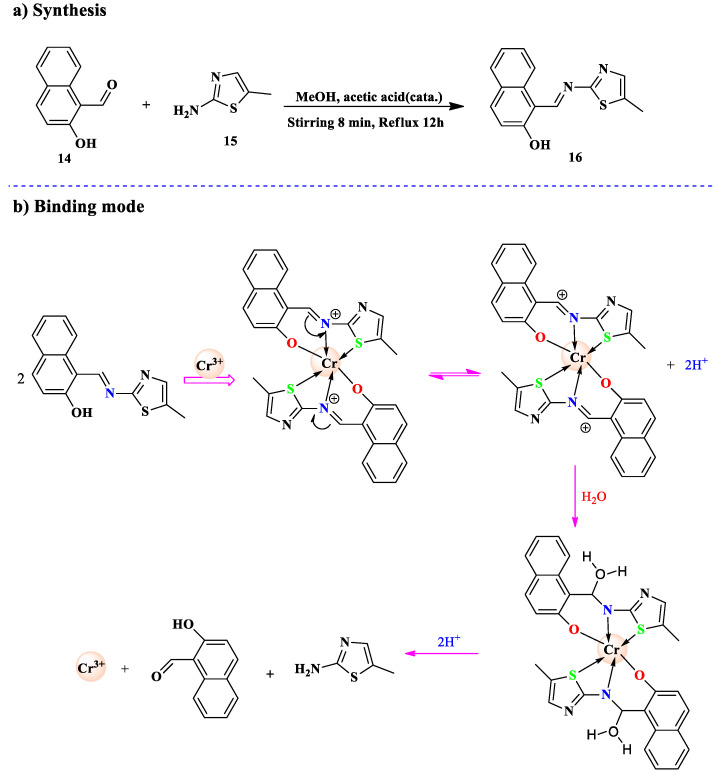
(**a**) Synthesis of Schiff-based chemosensor **16**, (**b**) proposed binding mode.

Chromium(III) modulates the metabolism of proteins, fats, and carbohydrates, and may boost the activity of specific enzymes. Primarily, it is essential for a variety of metabolic activities [74]. As an important micro-nutrient that is vital for preserving general human health and well-being, chromium(III) ions perform as an insulin potentiator, strengthening its beneficial effects [75]. On the other hand, high concentrations of chromium(III) ions are potentially damaging, disrupting cellular components and cellular structural framework, which might result in genetic abnormalities and even cancer risks [76]. In recent years, chromium(III) has been extensively released into the environment through many industrial processes, such as electroplating, leather tanning, mining, textile dyeing, and the manufacturing of inorganic chemicals. This has resulted in considerable environmental high contamination and has turned into an acute problem [77]. In the meantime, concerns about the harmful environmental impacts of chromium are still prevalent in both agricultural and industrial domains. This underlines the significance of more study concerns and the development of reliable detection approaches, particularly for Cr(III) ions in aqueous systems, with the goal of resolving these persisting environmental problems [78]. Lately, a series of products known as colorimetric and fluorescent chemosensors has gained traction or popularity in the sensing world for their capability to recognize and detect metal ions. These compounds provide a visible and highly sensitive response via color or fluorescence difference. Cr(II) metal ions are also first-row transition metal ions with *d*^4^ electronic configuration. They prefer N/O donor ligands. Due to its large size and moderate charge density, ligands that can provide multiple coordination sites help in their stabilization. They are a desirable option for sensing real world applications due to modification [79]. Although colorimetric and fluorescent organic chemosensors have demonstrated enormous potential in the detection of Cr(III) ions, there are still two main obstacles to overcome. Firstly, the waste of the majority of organic chemosensors is not soluble in pure water, which is vital for biological applications. Secondly, the currently available chemosensors often exhibit low selectivity for Cr(III) ions and are susceptible to interference from other metal ion species, potentially leading to false positive results [80]. Due to this, researchers have continuously designed and modified chemosensors with excellent selectivity, sensitivity, and solubility in water samples. Concerning their potential for effectiveness as ligands, tiny organic molecules with N, O, and S atoms, specifically Schiff bases, have appeared as the most promising candidates. These organic compounds are water-soluble due to their very small size and heteroatom existence, and they showed high selectivity, sensitivity, and affordable cost. Schiff bases are emerging choices for chemosensing applications as they effortlessly interact with various metal ions and resulting identifiable analytical signals for study [81]. S. Khan, M. Muhammad, and their colleagues effectively synthesized a chemosensor using a multi-step procedure. 2-hydroxy-1-naphthaldehyde **14** was first dissolved in CH_3_OH, and then a small amount of glacial acetic acid was added to serve as a catalyst. The reaction mixture was vigorously stirred for 8 min, a 2-amino thiazole methanol **15** solution was introduced to it, and the reaction mixture was refluxed for 12 h. The resultant mixture was cooled down to room temperature, and an 86% yield of the intended product **16** was obtained (Scheme 4). Purification was performed via recrystallization of the crude product from methanol [82].

**Figure 4:**
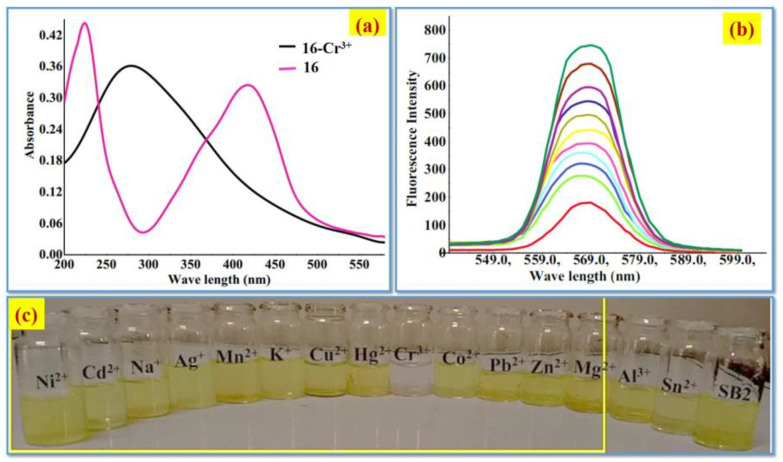
(**a**) Color changes in chemosensor **16** (2 µg mL^−1^) after the addition of 1.0 equivalent of different cations; (**b**) the fluorescence enhancement of chemosensor **16** (2 µg mL^−1^) in the MeOH–Water system (1/9 *v*/*v*) after the addition of 0.1–3 µg mL^−1^ of Cr^3+^ ions; (**c**) UV–Vis absorption spectra of chemosensor **16** and **16**-Cr^3+^. Copied from ref. [82] with permission from Springer, copyright 2022.

In the presence of various cations, including Na^+^, Mg^2+^, Ag^+^, Cr^3+^, Zn^2+^, Pb^2+^, Ni^2+^, K^+^, Mn^2+^, Hg^2+^, Co^2+^, Sn^2+^, Cu^2+^, Cd^2+^, and Al^3+^, the UV–Vis absorption studies of **16** were examined. The chemosensor **16** displayed two absorption bands centered at 220 and 422 nm (Figure 4a). Fluorescence spectra of chemosensor **16** were recorded in the presence of various cations in an aqueous medium (MeOH/H_2_O, 1:9 *v*/*v*) in order to examine the sensing capabilities of the chemosensor. Due to photo-induced electron transfer (PET) from the azomethine group (–C=N–) to the naphthalene fluorophore, free chemosensor **16** showed a modest fluorescence emission at 568 nm (λ_ex_ = 279). A remarkable turn-on response was observed upon the addition of Cr(III) ions to **16** (Figure 4b). Using various monovalent, divalent, and trivalent cations, such as Na^+^, Mg^2+^, Ag^+^, Cr^3+^, Zn^2+^, Pb^2+^, Ni^2+^, K^+^, Mn^2+^, Hg^2+^, Co^2+^, Sn^2+^, Cu^2+^, Cd^2+^, and Al^3+^, the colorimetric analyses of chemosensor **16** were carried out in aqueous media. The aqueous solution of **16** exhibited a yellow color. The addition of various cations did not significantly alter the color of **16** in aqueous environments, with the exception of the Cr(III) ion. When Cr(III) ions were added, the color changed dramatically from yellow to colorless (Figure 4c).

**Scheme 5:**
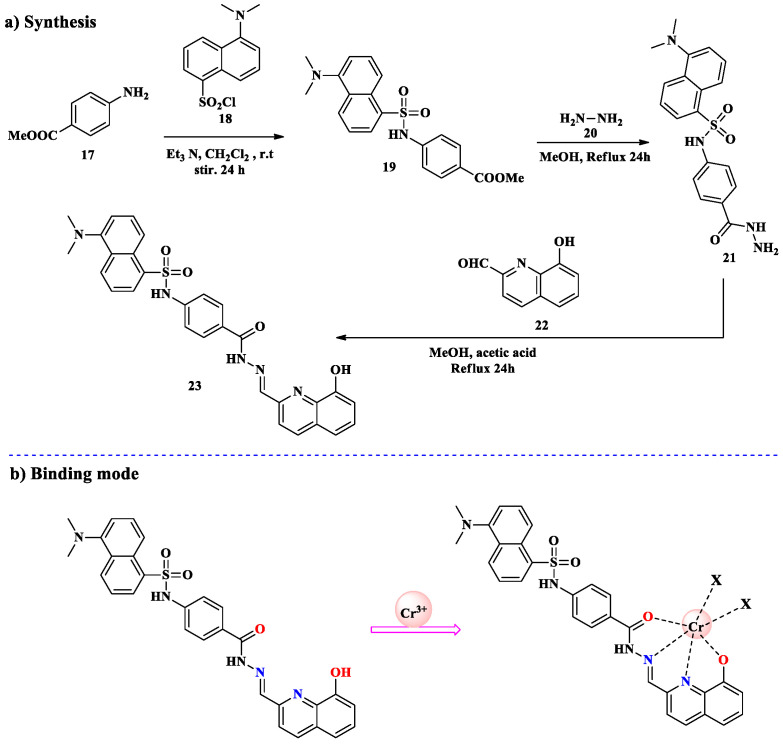
(**a**) Synthesis of Dansyl-based fluorescent chemosensors **23**, (**b**) proposed binding mode.

H. Wu and P. Zhou, along with their coworkers, developed a novel chemosensor with a tetradentate metal-binding moiety composed of 8-hydroxyquinoline-carboxhydrazone, which interacts with Cr^3+^ to form a complex. A solution of methyl 4-aminobenzoate **17** and trimethylamine in dichloromethane was added dropwise to a solution of 5-dimethylamino-1-naphthalenesulfonyl chloride (dansyl chloride, **18**) in dichloromethane. The reaction mixture was stirred at room temperature for 24 h. The desired product **19** was washed with water, and the organic layer was dried using MgSO_4_. The resulting yellow solid was recrystallized from ethyl acetate and used immediately in the subsequent step. The yellow solid product was then combined with hydrazine hydrate **20** in CH_3_OH and refluxed for 24 h. After solvent evaporation, the product was mixed with appropriate aldehydes **22** dissolved in methanol. Acetic acid was added dropwise to the reaction mixture, which was refluxed for another 24 h. After cooling, the crude mixture was filtered, yielding the desired yellow solid **23** (Scheme 5). A metal-binding moiety of 8-hydroxyquinoline-carboxhydrazone tetradentate is present in Sensor **23** and forms a 1:1 complex with Cr^3+^. Additionally, 23 showed a considerable increase in fluorescence but a much-decreased quantum yield after Cr^3+^ binding in an aqueous solution. A Job plot of the 23-Cr^3+^ fluorescence spectra, with the inflection point at around 0.5, which corresponds to [(23 − 2H) + Cr]^+^ in the ESI-MS spectrum of a combination of 23 and Cr^3+^, further verified the 1:1 binding mode [79].

**Scheme 6:**
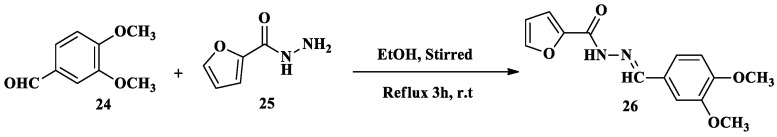
Synthesis of Schiff-based chemosensor **26**.

A.Abbasi and M. Shakir synthesized a chemosensor by adding methoxybenzaldehyde **24** with 2-furoic acid hydrazide **25** in EtOH at 25 °C. After about three hours of refluxing the solution mixture, a yellow-colored solution was obtained; from that, gradual solvent evaporation produced an off-white-colored crystalline precipitate including Schiff-based product **26** (Scheme 6). Cr(VI) effectively suppressed the fluorescence of chemosensor 26 through the primary and secondary inner filter effects. Cr(VI) has a detection limit of 0.175 μM and a linear recognition range of 1 μM to 500 μM. The addition of L-ascorbic acid in the concentration range of 10 μM to 390 μM with a LOD of 2.46 μM successfully turned on the chemosensor–Cr(VI) solution’s switched-off fluorescence. The reduction of Cr(VI) to Cr(III) by L-ascorbic acid is the mechanism suggested for the fluorescence turn-on of quenched fluorescence of the Cr(VI)–chemosensor solution of ascorbic acid. This process eliminated both primary and secondary inner filter effects and allowed the fluorescence of chemosensor 26 to return. It was discovered that the technique described in this study was successful in identifying Cr(VI) in groundwater and tap water, ascorbic acid in human serum, and vitamin C tablets [83].

### 2.2. Mn^2+^ Ion Detection

**Scheme 7:**
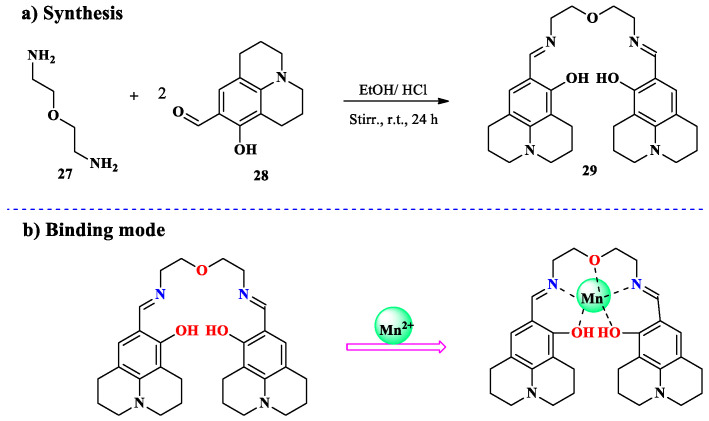
(**a**) Synthesis of chromogenic chemosensor **29**; (**b**) proposed binding modes.

The development of chemosensors is greatly facilitated by transition metal ions, such as Fe, Zn, Co, Cu, and Mn, due to their significant biological roles and the harmful environmental effects of their excess presence. Manganese (Mn) is one of the vital trace metals found in all living things and is involved in several important biological processes. Understanding its biological relevance and minimizing its environmental effects relies upon its identification and tracking processes [84,85]. Manganese is frequently employed in biological research, facilitating chemists to explore a broad range of biological processes and mechanisms due to its versatility and significant role as a cofactor in several enzyme groups and the evolution of the photosynthetic oxygen evolution process [86,87]. Manganese(II) ions have demonstrated substantial potential in their high-spin state, as a contrast reagent for magnetic resonance imaging (MRI), sparking passion in the neurobiology research field as a powerful tool for amplifying picture quality and studying brain function [88]. Because Mn^2+^ ions can be harmful to health, precise stimulation is crucial in neurobiology. Monitoring manganese ions level is important. Despite being somewhat less toxic, long term exposure even for short periods of time can trigger serious neurological consequences like movement or motor disorders, mental health disturbances, memory loss, and indication Parkinson’s-like symptoms [89]. Given their simpler properties, it is essential to design Mn^2^⁺-selective chemosensors that are capable of distinguishing Mn^2^⁺ from other transition metal ions, particularly Ca^2^⁺. This is crucial because Mn^2^⁺ and Ca^2^⁺ share the same cellular transport pathways, requiring precise detection and identification of these ions [90,91,92]. In addition to their amazing affinity for transition-heavy metal ions, the N atom in the Schiff bases’ azomethine (C=N) double bonds render them important chelating agents, as well as suitable potential sensors for identification and binding to metal ions [93]. Schiff bases are excellent or ideal choices for designed metal ion complexes and chemosensors because of their well-known strong binding features, with a range of applications, such as anticancer potential. This demonstrates the promising potential of Schiff bases in medicinal chemistry, biological research area [94], antioxidant properties, and appealing electronic and photophysical characteristics, ideally suitable for sensors, and imaging agents [95]. Fluorescent Schiff-based derivatives offer great potential as optical chemosensors for metal ions. Due to their capability to form complexes with metal ions and their unique coordination, stability, fluorescent attributes, and biological activities, symmetrical Schiff bases are attractive for a variety of purposes [96]. One major issue is the controlled synthesis of Schiff-based derivatives along with metal complexes. So many parameters, such as the ligand’s chemical structure, pH, the metal-to-ligand ratio interaction, reaction temperature, solvent medium system, and the metal’s optimal coordination geometry, all influence how well they self-assembled. These parameters affect the complex’s coordination geometry structure; thus, it is crucial to achieve the best synthesis conditions. Therefore, precisely and controllably designing these compounds is a difficult process that requires careful understanding of these aspects [97]. The field of designing fluorescence chemosensors with good selectivity and sensitivity to metal ions is a competitive and important one, with potential use in materials science, healthcare, and environmental monitoring processes [98]. Researchers strive to develop new metal ion chemosensors with greater sensitivity, selectivity, and reliability, even though there are multiple easily accessible commercial strategies available. This ongoing endeavor aims to meet all of the demands resulting from metal ions’ prevalence in living organisms and their huge importance in a range of biological operations [99]. A novel chemosensor was synthesized by Yeon Joo Lee, Chaejin Lim, and their team utilizing the condensation reaction of 2,2-oxybis(ethylamine) and 8-hydroxyjulolidine-9-carboxaldehyde. An ethanolic solution of 8-hydroxyjulolidine-9-carboxaldehyde **27** was made for the sensor by adding two drops of HCl, then gradual addition of 2,2-oxybis(ethylamine) **28** in EtOH (Scheme 7). Methylene chloride in methanol eluent was used in column chromatography to purify the obtained crude product **29** that resulted after 24 h of elimination of solvent under vacuum. This sensor **29** identified Mn^2+^ ions in MeCN-buffer solution by seeing a noticeable color shift from colorless to yellow [100].

**Figure 5:**
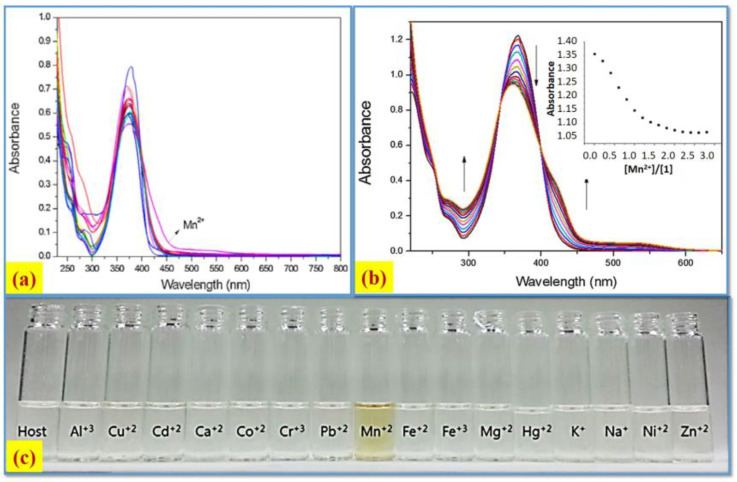
(**a**) The chemosensor **29’s** absorbance spectrum shifts in response to its exposure to various metal ions; (**b**) UV–Vis spectra of sensor 29 (30 M) upon the addition of Mn^2+^ in MeCN buffer solution (10 mM HEPES, pH 7.4, 9:1, *v*/*v*); (**c**) the presence of metal ions triggers an obvious color change in the chemosensor **29**. Copied from ref. [100] with permission from Elsevier, copyright 2014.

Using nitrate salts of several metal ions, including Ag^+^, Al^3+^, Ca^2+^, Cd^2+^, Co^2+^, Cr^3+^, Cu^2+^, Fe^2+^, Fe^3+^, Hg^2+^, Ga^3+^, In^3+^, K^+^, Mg^2+^, Mn^2+^, Na^+^, Ni^2+^, Pb^2+^, and Zn^2+^, in MeCN-buffer solution (9:1, *v*/*v*, 10 mM HEPES, pH 7.4) at room temperature, the chromogenic sensing ability of **29** was tested. As shown in Figure 5, While other metal ions produced little to no alterations, only the Mn^2+^ ion caused a noticeable spectrum shift and an immediate color change from colorless to yellow. The second colorimetric chemosensor used to detect Mn^2+^ in aqueous solution is this one. Significantly, this finding suggests that sensor **29** may be employed as a “naked-eye” Mn^2+^ ion sensor in aqueous conditions.

**Scheme 8:**
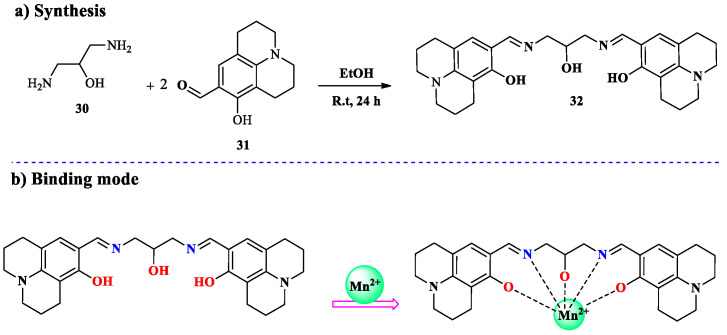
(**a**) Synthesis of Schiff-based chemosensor **32**, (**b**) proposed binding mode.

Two chemists K. B. Kim and G. J. Park have synthesized a novel Schiff-based chemosensor **32** containing a julolidine moiety. These can detect manganese(II) ions in aqueous solutions by establishing visible color changes. When this novel sensor interacts with Mn^2+^ ion, it shows a noticeable shift from light yellow to orange color. This color change can be identified with the naked eye. Chemosensor **32** was effectively synthesized by a straightforward coupling reaction of 1,3-diamino-2-propanol **30** and 8-hydroxyjulolidine-9-carboxaldehyde **31** (Scheme 8). The chemosensing characteristics of 32 were further examined by performing a UV–Vis titration of sensor **32** with Mn^2+^. The highest absorbance at 392 nm was rapidly reduced as increasing quantities of Mn^2+^ were added to a solution of 32. Three additional absorbances at 348 (ε = 3.4 × 10^4^ M^−1^ cm^−1^), 418 (ε = 3.2 × 10^4^ M^−1^ cm^−1^), and 532 nm (ε = 4.2 × 10 M^−1^ cm^−1^) emerged concurrently. These coefficients are too large to give Mn-based *d*-*d* transitions, they must give manganese to ligand-based transitions. Thus, the metal-to-ligand charge transfer (MLCT) may be responsible for the color shift from light yellow to orange in the presence of Mn^2+^. The development of a single species between sensor **32** and Mn^2+^ was shown by the obvious observation of the different isosbestic spots at 354 nm and 413 nm [101].

**Scheme 9:**
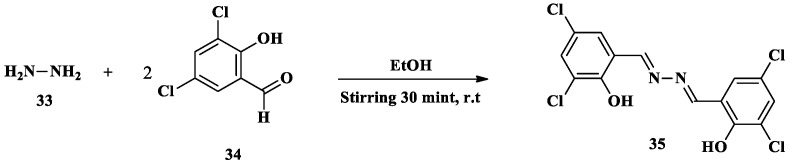
Synthesis of Schiff-based chromogenic sensor **35**.

V. Raju, R. S. Kumar, and their associates designed a novel chemosensor **35** by reacting hydrazine **33** with 3,5-dichloro-2-hydroxybenzaldehyde **34** in a simple one-step process. 3,5-dichloro-2-hydroxybenzaldehyde **34** was added in EtOH and then added drop by drop to hydrazine hydrate in a flask. Upon stirring the mixture for about half an hour, a precipitation started to appear. After that, 100% alcohol was used to filter and recrystallize the precipitate to obtain the intended product **35** (Scheme 9). Using a variety of metal ions, the sensing capabilities of sensor **35** were investigated using spectrofluorometric, UV–Vis, and optical tests in THF:H_2_O (1:1, *v*/*v*) media. Thus, when Mn^2+^ interacted with the original colorless solution of **35**, it became yellow, confirming the visual test; however, other ions did not react in natural light. Spectrophotometric techniques are used to examine the photophysical characteristics of **35**-Mn^2+.^ When clean **35** interacts with Mn^2+^, it shows a strong absorption band at 365 nm and a new absorption band at 425 nm. In contrast, other metal ions showed no emission characteristics. Additionally, Job’s plot was used to study the formation 3:2 binding stoichiometry between **35** and Mn^2+^, and the Benesi–Hilderbrand (BH) technique was used to measure the association constant. The WHO’s recommendations for metal ions in drinking water are significantly higher than the detection limit of **35** for Mn^2+^, which was determined to be 3.6 × 10^−7^ M. There is strong evidence from NMR and theoretical studies that **35** exhibits coordination behavior with Mn^2+^ [102].

### 2.3. Fe^3+^ Ion Detection

**Scheme 10:**
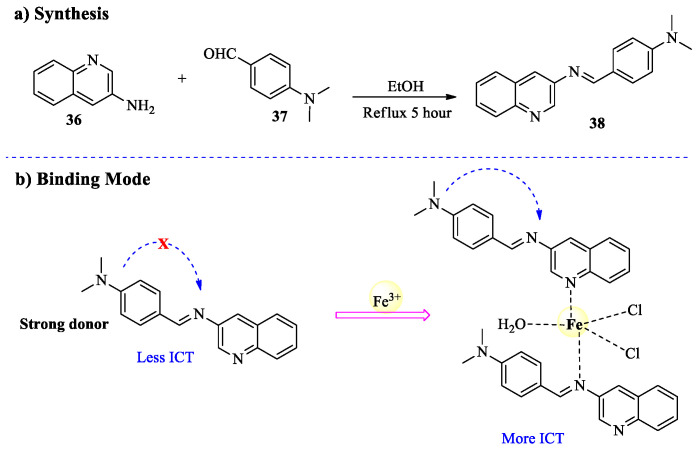
(**a**) Synthesis of DBQA Schiff-based sensor **38**; (**b**) proposed binding mode.

Of all the metal ions, iron is an essential component and one of the heavy metals found in the human body. It is believed that iron is essential to several physiological functions [103] as iron’s redox-active form catalyzes the generation of highly reactive oxygen species and can cause a variety of health issues. Excessive iron supplementation may be detrimental to the biological system. Consequently, the identification and study of Fe^3+^ has emerged as a difficult problem in environmental research and biomedicine in recent years. Now a days, the advanced development of chemosensors for Fe^3+^ detection has been quite fascinating. Since iron functions as hemoglobin, the primary oxygen carrier in all tissues, and since it supports the movement of electrons as cytochromes, iron is necessary for all biological systems [104]. Both the deficiency and excess of iron would result in various health conditions such as hemochromatosis, Alzheimer’s disease, and anemia (blood insufficiency), which may damage or even kill oxygen-dependent organs [105,106,107,108]. Through numerous kinds of agricultural and industrial processes, Fe^2+^ ions have major effects on crop yields, water body quality, and industrial operations. Hence, it is now crucial to efficiently track and accurately identify Fe^2+^ ions in biological, environmental, agricultural, and industrial resources [109,110]. Researchers may find ratiometric analysis more attractive as the ratio of the two emission intensities may be used to calculate the concentration of Fe^2+^ ions and be eligible for an automatic reimbursement for environmental effects and stability under fluorescence [106,111]. As a reference signal, one of the fluorophores must respond to a fluctuation in Fe^3+^, whereas the other is intended to be insensitive [112]. The offered chemosensor includes a highly selective ratiometric fluorescent sensor for Fe^3+^ detection and quantification [113,114,115]. Mohd Mustufa et al. synthesized a novel-N-(4-(dimethylamino) benzylidene) quinolone-3-amine (DBQA) fluorescent chemosensor. A heated ethanolic solution of 3-aminoquinoline **36** was added drop by drop to an ethanolic solution of 4-(dimethylamino)benzaldehyde **37** in order to develop the said chemosensor (DBQA). The reaction mixture was refluxed with agitation for five hours. After cooling, the greenish-black product was isolated by filtration. The product was recrystallized using ethanol, forming needle-shaped crystals that dried to a greenish-black tint. This protocol gave 75% yield of the final product (Scheme 10). By employing the synthesized chemosensor (DBQA) **38**, Fe^3+^ ion was efficiently detected. With the successive addition of the Fe^3+^ ion, the fluorescence and UV–visible spectra of (DBQA) demonstrated ratiometric activity and a bathochromic shift, exhibiting excellent sensitivity as well as the selectivity of the Fe^3+^ ion over other transition metal ions [116].

**Figure 6:**
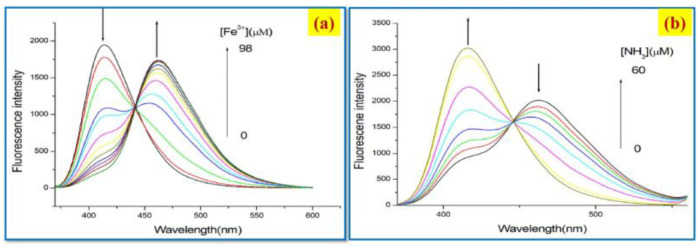
Fluorescence spectra of chemosensor **38** (**a**) DBQA (30 μM) in DMSO:H_2_O (*v*/*v*, 1:4, pH = 7.1) λ_ex_ = 290 nm simultaneous addition of 0–98 μM of Fe^3+^; (**b**) DBQA + Fe^3+^ (98 μM) in DMSO:H_2_O (*v*/*v*, 1:4, pH = 7.1) λ_ex_ = 290 nm successive addition of 0–60 μM of NH_3_. Copied from ref. [116] with permission from Elsevier, copyright 2023.

The mechanism of intramolecular charge transfer (ICT) allows DBQA to interact maximally with Fe^3+^ ions. This makes them an intriguing option for ratiometric fluorescence chemosensors, which are employed in semi-aqueous settings to detect metal ions [117]. Concerning DBQA’s selectivity for various metal ions, in the DBQA fluorescence spectrum, only Fe^3+^ demonstrates a ratiometric response when compared to other engaging metal ions. In the presence of more competitive metal ions, **38** was added to a DMSO:H_2_O solution to perform the competition assays. The other analyzed metal ions showed no noticeable ratiometric interaction when Fe³⁺ ions were detected. Figure 6 shows the emission spectra of the chemosensor (BQA). The emission at λ_em_ = 413 nm in DMSO:H_2_O (*v*/*v*, 1:4, pH = 7.1) at 25 °C led to the excitation wavelength to be set at 290 nm for the emission investigation. The behavior of the chemosensor (DBQA) towards Fe^3+^ and NH_3_ in a semi-aqueous liquid (DMSO:H_2_O) is demonstrated in this work. When varying amounts of Fe^3+^ were added, the emission intensity at 413 nm fell as the wavelength increased. A new peak at 463 nm with an isosbestic point at 446 nm was noticed as a result of the apparent ratiometric fluorescence change. When NH_3_ was added, the ratiometric response of DBQA with Fe^3+^ could be regained. Fe^3+^ would most likely be eliminated by NH_3_ as an ammonium iron complex.

**Scheme 11:**
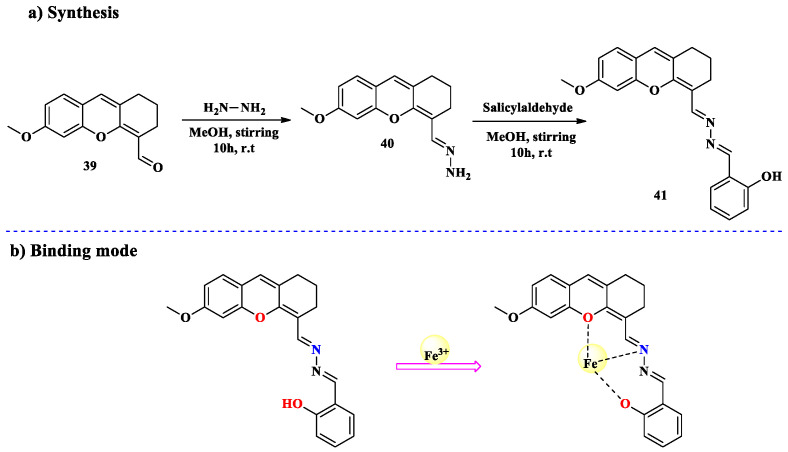
(**a**) Synthesis of bis-Schiff-based fluorescent sensor **41**, (**b**) proposed binding mode.

B. Li, X. Gu, and their colleagues successfully synthesized a novel chemosensor for detecting Fe^3+^ ions. The synthesis procedure involved several steps. Hydrazine hydrate was added after 6-Methoxy-2,3-dihydro-1H-xanthene-4-carbaldehyde **39** had dissolved in MeOH. After stirring the reaction mixture for ten hours, a crimson-red precipitate formed. The precipitate was filtered, and the solid was dissolved in MeOH, with the solvent removed by rotary evaporation. An orange-colored solid **40** was obtained after chilling the residual solution in a refrigerator. The hydrazinolysis product **41** was then dissolved in MeOH, mixed with o-hydroxybenzaldehyde, and stirred for 10 h at room temperature (Scheme 11). This led to the formation of an orange-red precipitate, which was filtered, purified by dissolving in MeOH, and dried by rotary evaporation. Finally, the residual solution was chilled, yielding the desired dark red crystals with a 66% yield. In a solution of MeCN-Tris (*v*/*v*, 2:1, 10 mM, pH 7.3), Sensor **41** continually detects Fe^3+^. Alongside the solution’s color shift from yellow to red, sensor **41** demonstrated a very sensitive and selective fluorescence “turn-on” for Fe^3+^ over other metal ions. Additionally, only F^−^ is detected by the resultant **41**-Fe^3+^ combination among the common different anions. Fluorescence and absorbance titrations, 1 H NMR titrations, HRMS, and theoretical computations were used to identify the bonding processes. Additionally, sensor **41** has been utilized to identify Fe^3+^ using test paper colorimetry and liquid colorimetry. Furthermore, the fluorescent imaging of Fe^3+^ in HeLa cells was performed sequentially off-on-off using sensor **41**. The photo-induced electron transfer (PET) process in sensor **41** may be activated by the detection of Fe^3+^. This is because the lone pair electron of the nitrogen atom should be transferred from the –C=N– group to the fluorophore unit, preventing the PET effect [118].

**Scheme 12:**
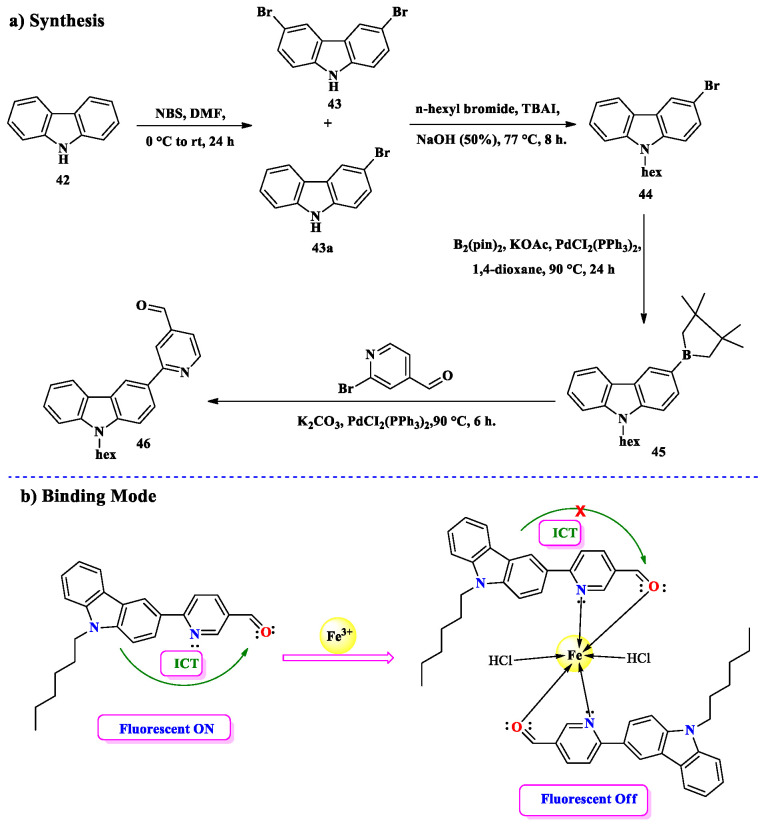
(**a**) Synthesis of carbazole-based chemosensor **46**; (**b**) proposed binding mode.

A. Battal, S. B. Kassa and their team developed a chemosensor for detecting Fe^3+^ ions. The compound was synthesized in four stages starting from carbazole. Firstly, carbazole **42** was brominated with N-bromosuccinimide (NBS) in dimethylformamide, producing a mixture of mono- and dibromo compounds. After crystallization, 3-bromocarbazole **43a** was obtained as white crystals in an acceptable yield. Next, 3-bromocarbazole was alkylated with 1-bromohexane in the presence of tetrabutylammonium iodide (TBAI) and 50% aqueous NaOH, yielding 3-bromo-9-hexylcarbazole **44** as a liquid in excellent yield after chromatography. The 3-bromo-9-hexylcarbazole **44** was then reacted with dichlorobis(triphenylphosphine) palladium(II) in 1,4-dioxane and bis(pinacolato)diboron, in the presence of potassium acetate (KOAc), to form the pinacol boronic ester **45**. Finally, (9-hexylcarbazole-3-yl)boronic acid pinacol ester underwent a Suzuki–Miyaura reaction with 2-bromopyridine-5-carbaldehyde in the presence of potassium carbonate and bis(triphenylphosphine)palladium(II) dichloride in tetrahydrofuran, yielding the intended compound **46** after multiple purifications by chromatography (Scheme 12). Then, a number of techniques were used to examine its sensing capabilities. The findings demonstrated that sensor **46** detects the Fe^3+^ ion more strongly than other interfering metal ions. Sensor **46’s** sensitivity and selectivity for Fe^3+^ were excellent for usage in applications. Additionally, it was noted that probe **46’s** fluorescence intensity dropped when aqueous Fe^3+^ ion solutions were applied to it using dimethyl sulfoxide (DMSO). The paramagnetic interaction between sensor **46** and Fe^3+^ ions is the cause of this situation (turn-off of emissions). Sensor **46’s** limit-of-detection (LOD) value was determined to be 1.38 nM. This value is insufficient to compete with its literary counterparts. In a real sample experiment, sensor **46** also showed a greater ability to detect Fe^3+^ ions than other ions in an actual medium. Consequently, it was determined that sensor **46** is a highly promising option for sensor technology [119].

### 2.4. Co^2+^ Ion Detection

Metal detection and recognition are essential because of their exclusive applications. In view of the many potential uses for these metals, chemists or researchers in related areas now face a huge difficulty in developing efficient techniques for identifying and detecting metal ions [120]. Among metal cations, cobalt ion (Co^2+^) comes out as a physiologically needed trace element, fundamental to many biological functions. Its flexibility has resulted in various implementations in a number of domains, like the commercial, industrial, and medical sectors, wherein its special qualities are taken advantage of to encourage advancement [121]. Moreover, cobalt ions require metal of many biological compounds, such as vitamin B_12_. Co^2+^ is also vital for the formation of hemoglobin and the metabolism of iron. Its presence is essential for preserving proper biological functioning since it aids in the regulation of iron metabolism and facilitates the formation of hemoglobin, further demonstrating its importance to human health and well-being [122]. On the other hand, long-term contact with cobalt metal can be damaging to human health and result in a number of toxicological side effects, such as digestive problems, damage to the liver, thyroid enlargement, respiratory illnesses like pulmonary disease and asthma, and even a disturbance in cardiac functions. Due to these probable health hazards, it is imperative to keep tabs on cobalt levels to protect people’s health and safety [123]. Therefore, for the purpose of ensuring the safety and quality of water resources and preventing possible adverse effects from cobalt ion contamination, the development of effective techniques for detecting cobalt ions in water samples is essential across a variety of industries and applications, including environmental monitoring, public health, and industrial applications [124]. Metal ions play vital functions in a number of domains such as environmental science, life sciences, and chemistry. There has been a lot of work directed into developing reliable and multifunctional chemosensors for detecting many types of metal ions [125].

**Scheme 13:**
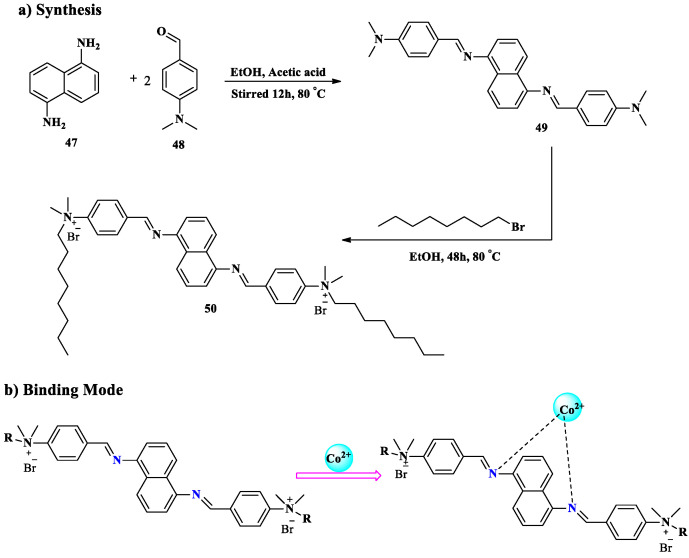
(**a**) Synthesis of naphthalene cationic Schiff-based chemosensor **50**; (**b**) proposed binding mode.

A. A. Abd-Elaal, A. Farag, and S. M. Tawfik successfully formed a unique chemosensor using a step-by-step procedure. After mixing 4-(dimethylamino)benzaldehyde **54** and naphthalene-1,5-diamine **47** in EtOH, with a few drops of glacial acetic acid as a catalyst, the reaction mixture was stirred for 12 h at 80 °C. This resulting yellowish solution was permitted to cool at room temperature, and after filtration and washing with cold diethyl ether, the precipitate was collected as a pale yellow powder containing 90% of intended compound **49**. After that, compound **49** was mixed in a nitrogen-filled round-bottom flask after being dissolved in EtOH. Upon adding octyl bromide, the reaction mixture was heated for 48 h at 80 °C. Then, the pale green precipitate produced was filtrated, washed with EtOH, and then recrystallized by diethyl ether to create a greenish powder that had a melting point of 312.5 °C and was 75% pure 4,4′-((1Z,1′Z)-(naphthalene-1,5diylbis(azaneylylidene))bis(methaneylylidene))bis(N,N-dimethyl-N octylbenzenaminium) dibromideoctylbenzenaminium) dibromide chemosensor **50** (Scheme 13). After adding the Co^2+^ ion, the intensity of quenched fluorescence was seen under a UV light, changing from blue to colorless; as a result, it may be used for detection with the naked eye. When sensor 50 interacts with Co^2+^ ions, its fluorescence intensity decreases, which other ions do not notice, indicating the sensor’s high selectivity. Sensor 50 reduces the LOD of CO^2+^ to 41 nM, which is below the WHO-approved detection limit. It is interesting to note that sensor **50** effectively demonstrated the utilization of the paper sensor for metal ion detection through color change analysis in each detection zone. Additionally, with sufficient recovery, sensor **50** can identify CO^2+^ ion contaminants in tap and river water. Their discovery of the application of a naphthalene cationic Schiff-based surfactant in optical sensing creates a research avenue for the development of a portable, low-cost sensor with enhanced sensitivity and specificity. This method detects threats in five minutes and does not involve any costly or intricate steps [126].

**Figure 7:**
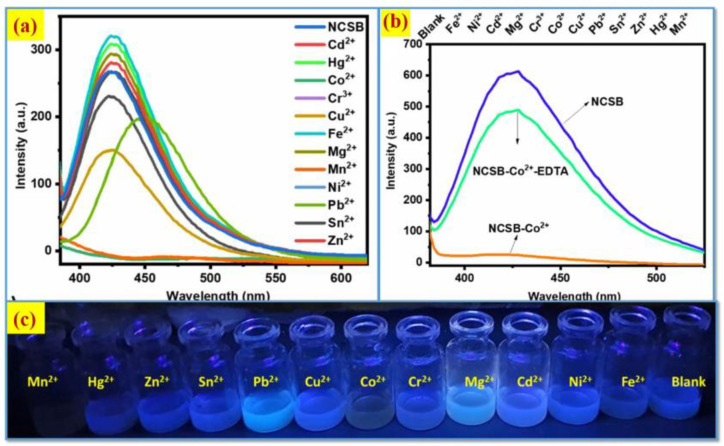
(**a**) Fluorescence spectra of **50** in the presence of various metal ion solutions (λex = 350 nm). (**b**) Fluorescence intensity changes that are reversible at **50** (250 µM) following the successive addition of EDTA and Co^2+^ ions in aqueous solution (λex = 350 nm). (**c**) Images of 56 aqueous suspensions (100 µM) with and without the addition of various metal ion solutions taken under 365 nm UV light illumination. Copied from ref. [126] with permission from Springer, copyright 2024.

To examine the selectivity of sensor **50** towards Co^2+^ spectrofluorometric titration experiments were performed. The fluorescence properties of **50** solutions with 12 different metal ions, including 1000 µM from each of Fe^2+^, Cu^2+^, Mg^2+^, Co^2+^, Ni^2+^, Zn^2+^, Cd^2+^, Hg^2+^, Pb^2+^, Sn^2+^, Cr^3+^ and Mn^2+^, are shown in Figure 7a. Surprisingly, Co^2+^ significantly reduced the fluorescence emission of sensor **50,** whilst other cations had no effect on the fluorescence intensity of sensor **50**. When Co^2+^ ions were added, sensor 50’s overall fluorescence intensity was quenched to 97.0%. This implied that substantial complexation between sensor **50** and the Co^2+^ metal ions through the imine (–C=N–) and quaternary ammonium (R_4_N^+^) groups is what drives the fluorescence quenching process. The solution for sensor **50** was incubated with 1 equivalent of a Co^2+^ solution before 1 equivalent of a disodium salt of ethylenediaminetetraacetic acid (Na_2_EDTA) solution was added to examine if the sensing performance of **50** is reversible. Following the injection of a Na_2_EDTA solution, sensor **50’s** original emission intensity from fluorescent **50**-Co^2+^ was nearly instantly restored (Figure 7b). This work demonstrated that the produced sensor may be easily regenerated and that the binding mechanism of sensor **56** with Co^2+^ is reversible. Furthermore, as seen in Figure 7c, the blue solution’s color changed to a colorless one with the addition of Co^2+^.

**Scheme 14:**
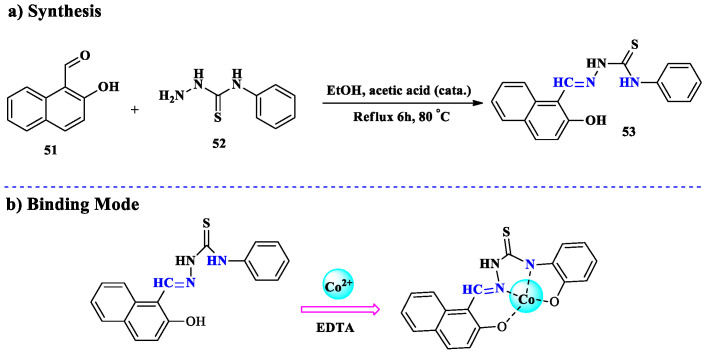
(**a**) Synthesis of thiosemicarbazide-based Schiff-based chemosensor **53**, (**b**) proposed binding mode.

Similarly, a chemosensor for detecting Co²⁺ ion was synthesized through a condensation reaction between 2-hydroxy-1-naphthaldehyde **51** and 4-phenylthiosemicarbazide **52**. In the synthesis procedure, a mixture of the two reactants in absolute ethanol and glacial acetic acid was refluxed for 6 h at 80 °C in the presence of an organic catalyst. The solid product was then cooled, filtered, and purified. After recrystallization from an EtOH-H_2_O mixture, chemosensor **53** was obtained with a high yield of 95% (Scheme 14) [127].

**Scheme 15:**
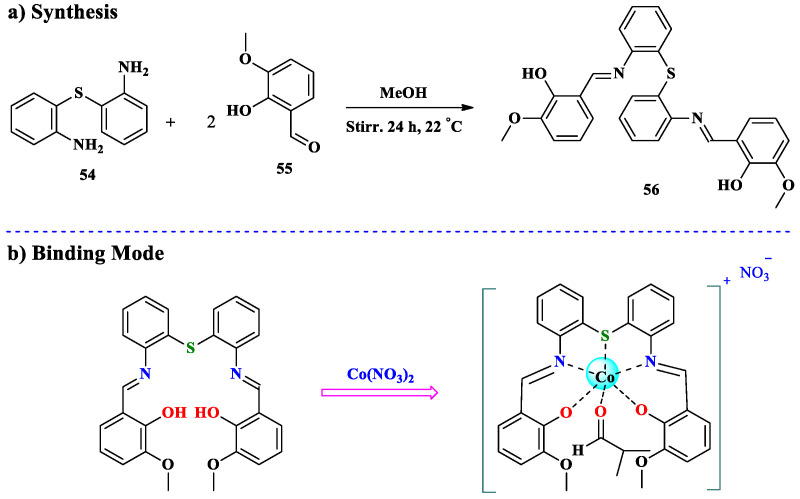
(**a**) Synthesis of chelated-type chemosensor **56**; (**b**) proposed binding mode.

P. A. Kim, H. Lee, and their team developed a chemosensor for detecting cobalt ions. They dissolved 2,2′-Thiodianiline **54** in methanol and then mixed in 2-hydroxy-3-methoxybenzaldehyde **55**. An orange precipitate appeared when the mixture was stirred regularly for the whole day at 22 °C. After filtration and washing with cold EtOH, the precipitates produced 82% of final product **56** (Scheme 15). A chelated-type colorimetric chemosensor **56** was designed for sensing Co^2+^ in an aqueous solution. Sensor **56** has modest detection limits (0.66 μM for Co^2+^) and can clearly detect Co^2+^ by changing color from colorless to yellow. Co^2+^ could be measured in water samples using TAMS. FT-IR, ESI-mass, UV–Vis measurements, and theoretical computations were used to illustrate the **56** to Co^2+^ sensing processes [128].

### 2.5. Ni^2+^ Ion Detection

Nickel is an incredibly shiny, malleable, and versatile ferromagnetic metal. It is an important component in the production of stainless steel and alloys resistant to corrosion because of its remarkable resistance to environmental corrosion. Moreover, because of its unique properties and resilience, nickel is useful in a variety of applications, such as electroplating, coin manufacturing, petroleum refining industries, textile production, printing, mold-making, and medicinal treatment operators [129]. The extraction and utilization of nickel from ores has grown due to increasing demand for metal. Nevertheless, the techniques used for both its extraction and application are not environmentally friendly. Numerous forms of environmental pollution such as air, water, and soil damage have been identified and recognized as a result of nickel levels exceeding permissible limits. This has adverse impacts on plant, animal, and human health, reinforcing the need for the nickel sector to switch to more environmentally friendly and more sustainable processes [130]. Devastating implications for the environment arise from nickel contamination of the soil and water, notably lower aquatic life production and lesser crop yields. In addition to harming the environment, nickel pollution has serious toxic effects on economic growth and food security, demonstrating the crucial importance of efficient mitigation measurements to safeguard our environment [131]. When airborne nickel metal ions are inhaled, they can enter the body promptly via the respiratory system, producing a prolonged intake and negative consequences later on. These adverse effects include allergic reactions on the skin, pulmonary fibrosis, cardiovascular damage, and a higher risk of cancer, such as kidney and nose cancer. Long-term exposure to nickel metal ions can have detrimental effects on health. That is why it is important to monitor exposure and put safety precautions in action [132]. Considering that nickel contaminants are perilous for the environment, tracking them is a key area of scientific interest. Though there are numerous instrumental procedures for monitoring nickel metal ions, they are sometimes highly expensive, time-consuming, and challenging to perform. In response, scientists have turned to molecular recognition structures as an alternative treatment. These frameworks lead to more accurate and reliable methods of nickel monitoring by supporting the creation of efficient chemosensors that can successfully detect Ni^2+^ metal ions in aqueous solutions with a high selectivity and sensitivity response [133]. The simplicity, ease of use, high sensitivity, and selectivity of fluorescent chemosensor-based research approaches have garnered much attention, making them a preferred choice over conventional analytical detection techniques [134]. The development of sensors with the best feasible selectivity, sensitivity, and rapid reaction time has been the primary objective of numerous research studies [135]. Multiple fluorescent chemosensors with amplified fluorescence signals have been intended to detect Ni^2+^ ions with higher sensitivity [136].

**Scheme 16:**
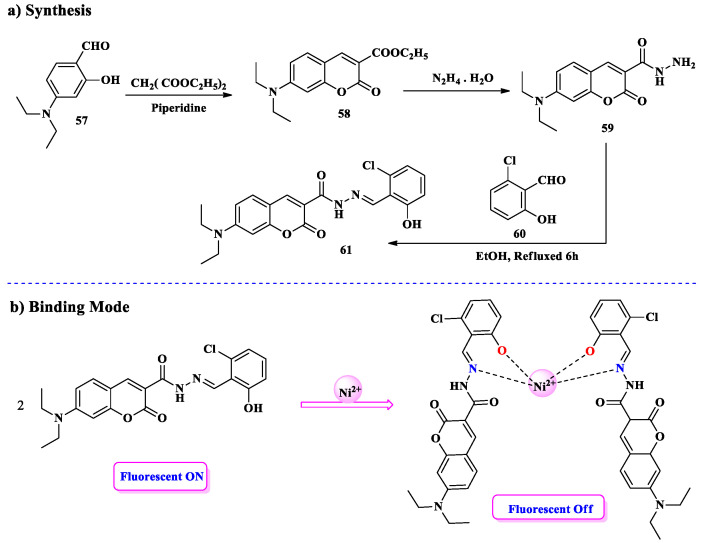
(**a**) Synthesis of acylhydrazone coumarin fluorescent chemosensor **61**; (**b**) proposed binding mode.

W. Lu and J. Chen, along with their coworkers, synthesized a chemosensor for the detection of Ni^2+^ ions. The experimental results revealed outstanding sensitivity to Ni^2+^ ions, efficient selectivity, and a significantly low detection limit. In order to design the required chemosensor, reactions of **59** and 2-chloro-6-hydroxybenzaldehyde **60** were refluxed in anhydrous EtOH for 6 h. The reaction mixture was then cooled to room temperature and washed with EtOH to yield the yellow solid product (Scheme 16) [137].

**Figure 8:**
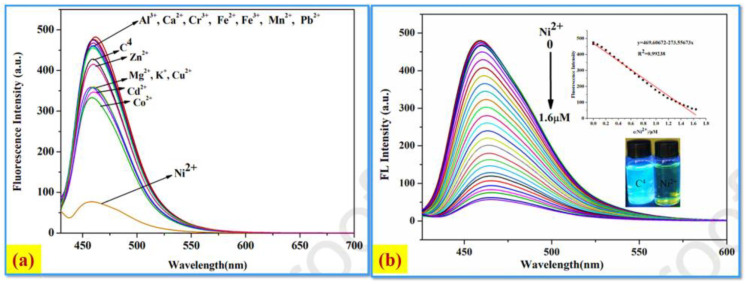
(**a**) Fluorescence spectra of sensor **61** in the presence of different metal ions (THF:PBS = 99:1, *v*/*v*, λex = 426 nm). (**b**) Standard curve for fluorescence emission intensity vs. Ni^2+^ concentration (λex = 426 nm). Insert: Color change induced by the addition of Ni^2+^ (1 equiv.) to the solution of **61** (**61** = 1 × 10^−6^ mol/L, THF:PBS = 99:1, *v*/*v*). Copied from ref. [137] with permission from Elsevier, copyright 2020.

The fluorescence was observed when a number of metal ions (Al^3+^, Ca^2+^, Cd^2+^, Co^2+^, Cr^3+^, Cu^2+^, Fe^2+^, Fe^3+^, K^+^, Mg^2+^, Mn^2+^, Na^+^, Ni^2+^, Pb^2+^, and Zn^2+^) were added to solution **61** to assess the selectivity of **61** towards Ni^2+^. There is a clear fluorescence response only to Ni^2+^. When Ni^2+^ was added, the solution’s color changed from brilliant yellow to dark yellow (Figure 8). Solution **61**’s fluorescence intensity was quenched by the addition of Ni^2+^. As seen in Figure 8a, fluorescence titration profiles of **61** were performed by increasing the concentrations of Ni^2+^ in the solution to assess the binding capacity of **61** to Ni^2+^. The fluorescence of **61** at 462.5 nm rapidly lost strength as the quantity of Ni^2+^ increased. According to LOD = 3σ/k, the concentration range of 1.3 × 10^−6^ to 1.6 μM, Ni^2+^ produced the lowest and most steady fluorescence signal and strong linearity in the fluorescence intensity range (R_2_ = 0.99238). A standard curve that showed a strong linear relationship between the concentration of Ni^2+^ ions and the fluorescence intensity was produced. This curve made it feasible to precisely measure Ni^2+^. The addition of Ni^2+^ ions resulted in a discernible color change in the chemosensor solution, providing a clue as to sensor **61**’s responsiveness or selectivity as well as its visual visibility (Figure 8b) [137].

**Scheme 17:**
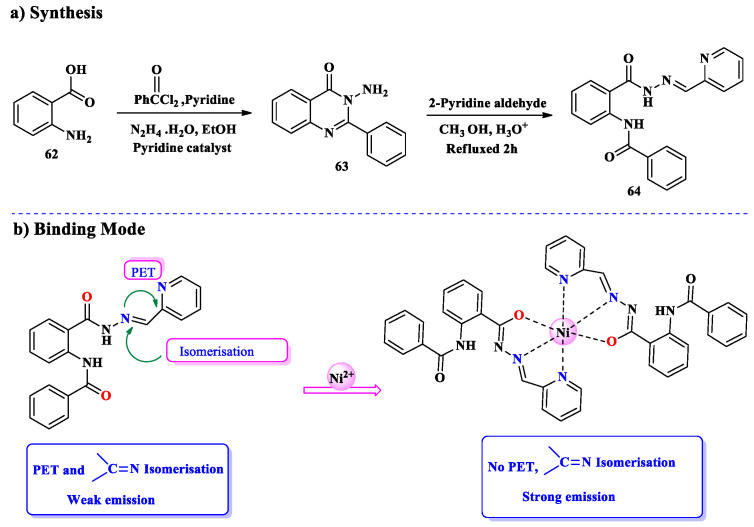
(**a**) Synthesis of Schiff-based chemosensor **64**, (**b**) proposed binding mode.

A. K. Manna, S. Chowdhury, and their collaborators efficiently prepared a novel versatile chemoreceptor that exhibited high potential as a highly sensitive and specific nickel ion sensor by displaying a selective ‘turn-on’ colorimetric and fluorometric response to Ni^2+^ ions in MeOH. The reaction of 3-amino-2-phenyl-4(3H)-quinazolinone **62** with 2-pyridine carboxaldehyde was performed in dehydrated MeOH. Glacial acetic acid was added as a catalyst to synthesize a Schiff-based chemoreceptor **63**. A crystalline solid appeared as the mixture was refluxed for 2 h in a dry air environment and then permitted to stand in the air for one day. After filtration, washing, and air drying, the solid product was obtained with 85% yield (Scheme 17). A colorimetric and fluorometric “turn on” response was displayed by sensor 64 when only Ni^2+^ ions were present in the methanol–Tris-HCl buffer solution (10 mM, pH 7.2, 1:1 *v*/*v*). The ESI-MS study further supported the Job plot analysis’s finding that the receptor forms a 2:1 complex with Ni^2+^ ions. Additionally, a single crystal of the **64**-Ni^2+^ complex was extracted. According to colorimetric and fluorometric calculations, the detection limit was 1.8 µM and 1.18 µM, respectively. These values are significantly lower than those suggested by the WHO drinking water standards. Chemosensor **64** was used for live cell imaging, smartphone-based analysis, molecular logic gate construction, and the recovery of tainted water samples [134].

### 2.6. Cu^2+^ Ion Detection

**Scheme 18:**
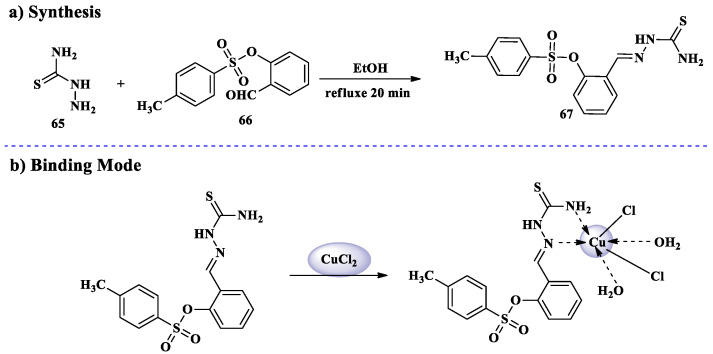
(**a**) Synthesis of CHMPMBS chemosensor **67**; (**b**) proposed binding mode.

Copper, the third most prevalent transition metal in the body, is necessary for a number of physiological functions. It performs a vital role in human digestion. Most of the time, it is probable that Cu^2+^ is always dangerous to microbes and marine life [138]. Cu^2+^ pollution has grown increasingly commonplace worldwide as a result of the growing usage of copper in appliances at home and in water pipes, industry, and agriculture. Analytical methods that are rapid, inexpensive, simple, and trustworthy are essential for detecting copper in drinking water and biological specimens, both qualitatively and quantitatively [139,140]. Cu^2+^ is a *d^9^* ion with a strong preference for N-donor ligands. It often adopts a square planar or distorted octahedral geometry, making selective binding possible through steric and electronic effects. It has been reported that colorimetric and fluorometric chemosensors have been designed to detect Cu^2+^ ions through changes in fluorescence intensity and color [141]. H. Hrichi and his coworkers synthesized 2-[(carbamothioylhydrazono)methyl-phenyl-4-methylbenzenesulfonate **67**, which is identified by NMR and FTIR spectroscopy and has selective colorimetric chemosensor characteristics for Cu^2+^ ions detection. In an aqueous medium with several competing cations present, such as Na^+^, K^+^, Mg^2+^, Al^3+^, Ag^+^, Ca^2+^, Fe^3+^, Fe^2+^, Co^2+^, Ni^2+^, Cr^3+^, Pb^2+^, Zn^2+^, Ba^2+^, and Cd^2+,^ the **67** chemosensor exhibited excellent sensitivity and selectivity toward Cu^2+^ ions. By combining Cu^2+^ and CHMPMBS in a 1:1 stoichiometry to form (CHMPMBS:Cu)complex, the chemosensor **67** showed a noticeable color shift from light yellow to green. This compound offers a quick and precise colorimetric test for Cu^2+^ ion detection with the naked eye. Chemosensor **67** was designed using standard protocol. Thiosemicarbazide **65** and 2-tosyloxybenzaldehyde **66** were added to and dissolved in ethanol and subsequently refluxed (Scheme 18). The precipitate was cooled at room temperature before being filtered and crystallized from the EtOH. The developed chemosensor **67** gave a distinctive color change from pale yellow to pale green. Sensor **67** (CHMPMBS) exhibits a rapid and accurate colorimetric assay for naked-eye detection of Cu^2+^ ions with a detection limit of 8.12 μM [142].

**Figure 9:**
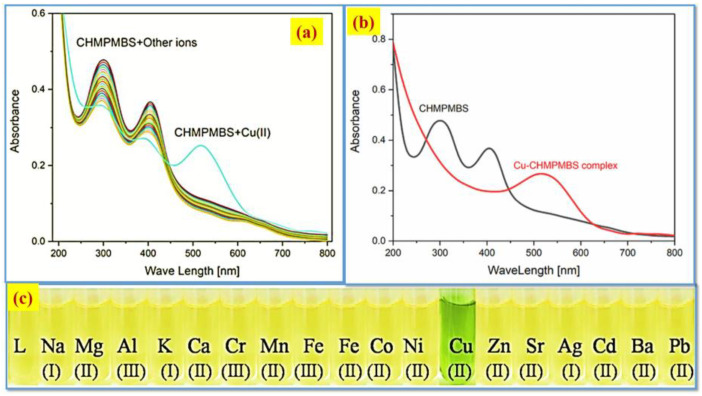
(**a**) The physical appearance of a new charge transfer band was the reason underlying the color shift in the **67**. The specific CHMPMBS 67 absorption bands vanished as a result of the paramagnetic Cu^2+^ interaction with CHMPMBS, and an entirely distinct broadband emerged at 515 nm. The concentration of the other ions was approximately double that of the Cu^2+^ ions. The intensity of the Cu^2+-^CHMPMBS complex’s absorbance is not substantially influenced by other metal ions; (**b**) color shift visible to the naked eye when 4 different cations are added to the CHMPMBS solution in DMF-water. (**c**) Absorption spectra of CHMPMBS. Copied from ref. [142] with permission from Springer, copyright 2023.

The cation recognition behavior of CHMPMBS sensor **67** with Na(I), Mg(II), Al(III), K(I), Ca(II), Cr(III), Mn(II), Fe(III), Fe(II), Co(II), Ni(II), Cu(II), Zn(II), Sr(II), Ag(I), Cd(II), Ba(II), and Pb(II) at pH = 6.5 was investigated using UV–Vis absorption spectroscopy. A new charge transfer band appeared, which resulted in the color shift of CHMPMBS. The distinctive CHMPMBS absorption bands vanished as a result of the paramagnetic Cu(II) interaction with CHMPMBS, and clear broadband emerged at about 515 nm (Figure 9a). By mixing 4.0 mL of different cations (50 µM, in H_2_O) with 1.0 mL of the CHMPMBS solution (50 µM, in DMF-water (1:1 *v*/*v*), the colorimetric ability of CHMPMBS was investigated. The absorption spectra of the CHMPMBS in water-DMF (1:1, *v*/*v*) solvent showed two distinct absorption bands at 290 nm and 405 nm, respectively, which correspond to the π–π* and n–π* electronic transitions (Figure 9b). The distinctive **67** absorption bands disappeared when the Cu^2+^ interacted with CHMPMBS, and new broadband, caused by d–d transitions, emerged at about 515 nm. Chemosensor **67** demonstrates the characteristics of being sensitive, selective, and qualitatively capable of detecting Cu^2+^ ions by displaying a notable color shift from pale yellow to pale green as a result of detecting Cu^2+^ ions (Figure 9c). There was no change in color in several other samples screened for cations [143].

**Scheme 19:**
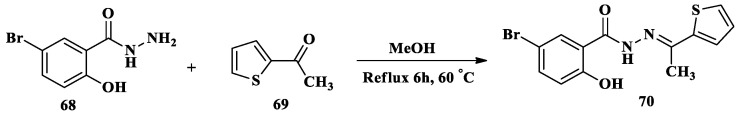
Synthesis of hydrazone-based sensor **70**.

S. A. Rupa synthesized a new chemosensor **70**. A solution of 2-acetyl thiophene **69** in CH_3_OH was mixed with a solution of 5-bromo-2-hydroxybenzohydrazide **68** in methanol. The resultant mixtures were agitated at 60 °C for six hours with continuous stirring. During the procedure, a yellow precipitate was produced, which was filtered out and vacuum-dried, producing a 47% yield (Scheme 19). Chemosensor **70** was shown to be insensitive to different metal ions; however, its sensing investigations showed a turn-on-enhanced fluorescence and colorimetric response toward Cu^2+^ ions. The binding stoichiometry of sensor **70** and metal ions is 2:1, according to the Job plots. Furthermore, the results of density functional theory (DFT) clearly suggested that chemosensor **70** might be employed as a potent colorimetric sensor for Cu^2+^ ion detection. Disk diffusion was used to assess the in vitro antimicrobial properties of sensor **70**, and the findings showed excellent antibacterial activity against *E. coli* [144].

**Scheme 20:**
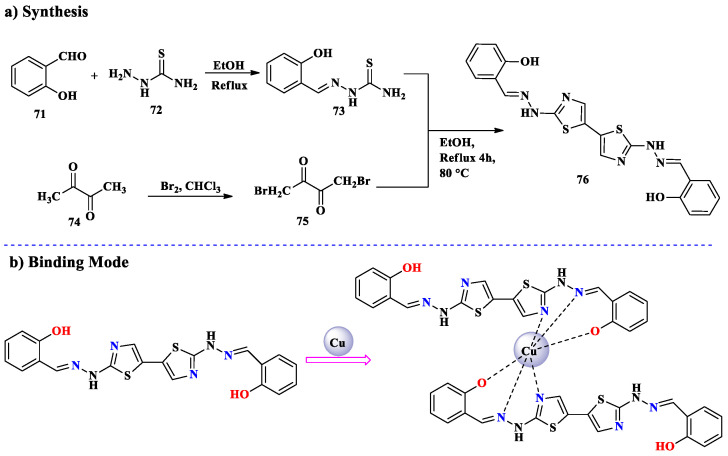
(**a**) Synthesis of Thiazolyl-hydrazone-based sensor **76**; (**b**) proposed binding mode.

Z. Jia, J. Wei, and their collaborators devised and manufactured a Cu^2+^-specific chemosensor probe called thiazolyl-hydrazone. Absolute ethanol was used to dissolve 2-(2-hydroxybenzylidene) hydrazine-1-carbothioamide **73** and 1,4-dibromobutanedione **75**. The reaction mixture was refluxed for four hours. The precipitate was filtered and recrystallized with DMF and ethanol. A brown-colored solid product produced a 53% yield (Scheme 20). With a binding stoichiometry of 1:1, a Cu^2+^-specific chemosensor **76** thiazolyl-hydrazone was formed, offering a tridentate chelate NNO coordination mode for Cu^2+^.When the sensor **76** solution binds to the Cu^2+^ cation, it quickly changes from colorless to yellow and develops a new, strong absorption band at 414 nm. This change is not induced by other metal cations. The detection limit for the naked eye is as low as 20 μM, whereas the analytical detection limit is 0.75 μM. Furthermore, at a detection limit of 7.2 μM, Cu^2+^ could quenched the emission signal of sensor **76** [145].

### 2.7. Zn^2+^ Ion Detection

**Scheme 21:**
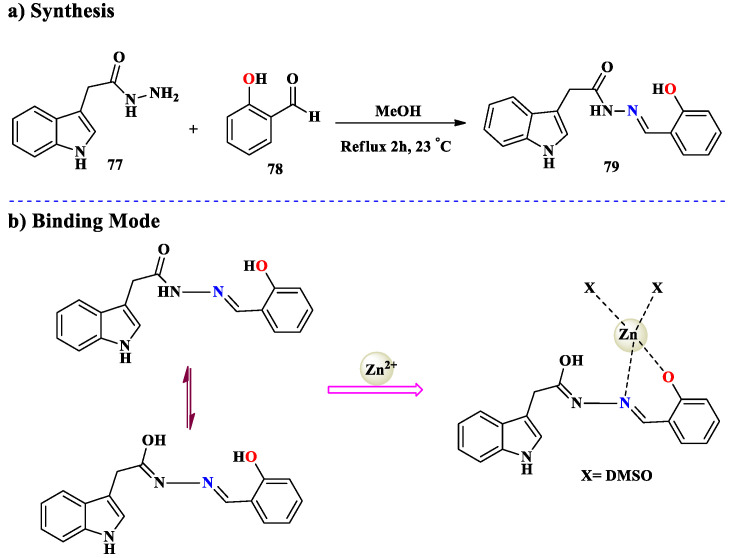
(**a**) Synthesis of indole-based fluorescent chemosensor **79**; (**b**) proposed binding mode.

Zinc ions are the second most frequent element in the human body. Numerous biological activities, including gene transcription, brain function, and immune system function, depend on the Zn^2+^ ion [146,147,148]. Even so, an overabundance of Zn^2+^ ions would upset the equilibrium of cellular functions, leading to neurodegenerative conditions like Parkinson’s and Alzheimer’s disease, diabetes, prostate cancer, Wilson and Menkes, etc. [149,150,151,152,153,154]. Furthermore, it is difficult to identify Zn^2+^ ions in biological systems because they are colorless in aqueous solutions, do not have unpaired electrons, and remain in the positive two-oxidation state. Zn^2+^ is a *d^10^* closed-shell ion with no ligand field stabilization energy. It prefers O/S/N donors, typically forming tetrahedral or octahedral complexes. According to this perspective, fluorescent sensors are effective instruments for tracking zinc ions as the selective adsorption of zinc ions on chemosensors can alter the fluorescence characteristics [155]. Consequently, there has been a lot of interest in various research on improving the sensitivity of fluorescent probes for Zn^2+^ ions. To detect Zn^2+^ ions, a fluorescent chemosensor based on indole (**79**) was developed. This exhibited an obvious rise in fluorescence upon interaction with Zn^2+^ ions. The World Health Organization’s approved criterion for Zn^2+^ detection using chemosensors was far lower than that chemosensor. This can accurately represent, identify, and measure Zn^2+^ in actual water samples and living organisms. Fluorescence and UV–visible spectroscopy have been used to show **79**-Zn^2+^ detection approach. Salicylaldehyde **78** and 2-(1H-indol-3-yl)acetohydrazide **77** were refluxed in MeOH for two hours at 23 °C to give [(E)-N0-2-hydroxybenzylidene)-2-(1H-indol-3-yl)acetohydrazide] **79** (Scheme 21). The detection limit (0.41 μM) of sensor **79** can quantify Zn^2+^ in real water samples. Moreover, it could identify and illustrate the presence of Zn^2+^ in zebrafish [156].

**Figure 10:**
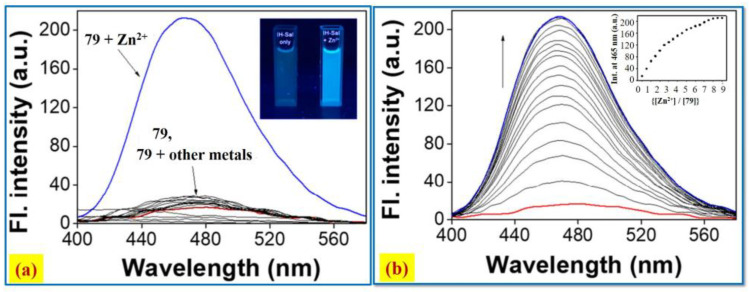
(**a**) Fluorescence titration of **79** with varying quantities of Zn^2+^; (**b**) fluorescence variations in **79** with variable metal ions and the fluorescence pictures of **79** and **79** + Zn^2+^ under UV light. Copied from ref. [156] with permission from MDPI, copyright 2021.

The fluorescence variance was observed using different metal ions in bis-tris buffer (Figure 10). There was no fluorescence emission from **79** directly. The Zn^2+^ ion interaction caused a noticeable increase in fluorescence at 465 nm (λex = 369 nm), along with an important Stokes shift. Out of all the chemosensors, **79** + Zn^2+^ had the biggest Stokes shift.

**Scheme 22:**
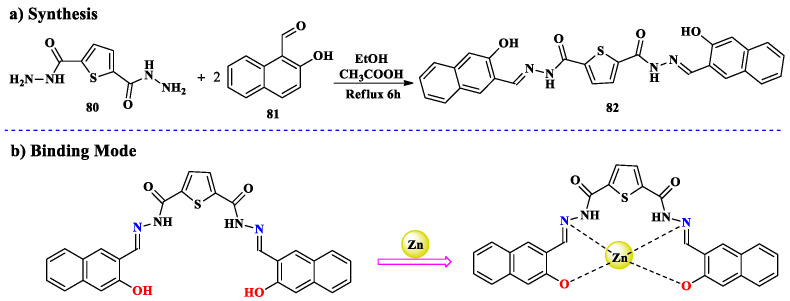
(**a**) Synthesis of Thiophene diocarbohydrazide-based sensor **82**; (**b**) proposed binding mode.

M. M. Mathew and A. Sreekanth developed a novel chemosensor **82** for detecting Zn^2+^ ions. They added 2-hydroxynaphthaldehyde **81** in EtOH with a stirred solution of thiphene-2,5-dicarbohydrazide **80**, followed by the addition of a few drops of glacial acetic acid as catalyst. The reaction mixture was then refluxed for 6 h, and the reaction progress was monitored using TLC. This resulted in the generation of a dark yellow precipitate (yield = 93%), which was collected via simple filtration and dried under vacuum (Scheme 22). Chemosensor **82** was discovered to be highly emissive upon complexation with the Zn^2+^ ion. In a DMSO/H_2_O (6:4 *v*/*v*) solution, this turn-on fluorescence recognition was prepared. After calculation, the binding constant was determined to be 1.15 × 10^4^ M^−1^. The limit of detection (LOD) was determined to be 1.51 × 10^−7^ M, which is significantly lower than the allowable amount of Zn^2+^ (70 μM) in drinking water. Chemosensor **82**-Zn^2+^ complex’s 1:1 binding stoichiometry was predicted by Jobs’ plot. By using a chelation-enhanced fluorescence mechanism, the observed fluorescent enhancement turn-on emission may be eliminated. The **82**-Zn^2+^ combination with EDTA was subjected to the appropriate INHIB-IT/IMPLICATION Logic gate behavior and reversibility tests [157].

**Scheme 23:**
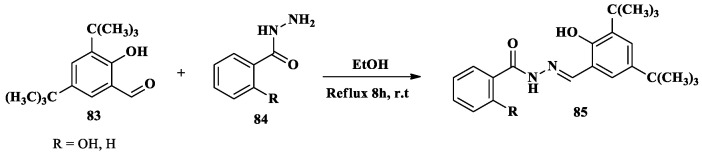
Synthesis of Arylhydrazone derivative as fluorescent sensor **85**.

X. Peng, X. Tang and others synthesized N-(3,5-di-tert-butylsalicylidene)-2-hydroxybenzoylhydrazine. It was detected as a Zn^2+^ fluorescence chemosensor. Salicyloyl hydrazide **84** was introduced to a stirred solution of 3,5-di-tert-butyl-2-hydroxybenzaldehyde **83** in EtOH. After eight hours of reflux, the reaction mixture was permitted to settle to room temperature. The resultant yellow precipitates **85** were filtered, cleaned with ethanol, and vacuum-dried. A yield of 80% of product **85** was obtained (Scheme 23). The addition of a single hydroxyl group to chemosensor **85** not only exhibits a robust interaction with Zn^2+^, but also sets this metal cation apart from its rivals, which include the potent Cd^2+^, Mg^2+^, and Ca^2+^ ions. The development of metal–ligand complexes was responsible for the rise in emission in the presence of Zn^2+^. Because of the high coordination of Zn^2+^ that would impose stiffness, the fluorescence response of **85** is improved by Zn^2+^-selective chelation by around 25-fold. One explanation might be that the substituent hydroxyl group improves the Zn^2+^ ion’s chelating capacity [158].

## 3. Conclusions and Future Perspectives

Transition metal ions, particularly the first-row ones, are common and serve a variety of biological purposes. A significant accumulation of these ions, however, may be harmful to one’s health. Therefore, it is crucial to accurately determine their spatial distribution both inside and outside of cells. The amplification of fluorescence by transition metal ions has potential applications beyond biological sensing. However, the majority of transition metal ions are paramagnetic and efficiently quench fluorescence. Optical chemosensors remain a highly active area of research, as evidenced by the papers reviewed. Future efforts should focus on developing and utilizing fluorescent probes that function effectively in pure aqueous solutions at physiological pH levels. This feature article aims to provide readers with a deeper understanding of various synthetic approaches of Aryl-substituted thiocarbohydrazide, thiosemicarbazide, thiazolyl-hydrazone, thiophene diocarbohydrazide, Arylhydrazone, indole, Schiff-based, carbazole, acylhydrazone coumarin chromofluorescent sensors, etc., and their mechanisms of action. We categorize colorimetric and fluorometric sensors for the detection of first-row transition metal ions, including Cr³⁺, Mn²⁺, Fe²⁺, Co²⁺, Ni²⁺, Cu²⁺, and Zn²⁺, while highlighting their ability to simultaneously detect multiple ions. The development of these sensors addresses critical challenges related to the rapid and straightforward detection of hazardous metal ions at trace levels in biological and environmental systems. As we proceed, it is evident that the synthesis of fluorescent chemosensors is far from finished [159]. Their capabilities could be further improved by emerging technologies like fluorescent-based sensors. Furthermore, point-of-care diagnostics and customized healthcare are being revolutionized by the incorporation of these sensors into portable devices [31,160,161]. This study presents a comprehensive overview of current methodologies, and the insights provided here aim to inspire further research to meet the evolving demands in the field of trace transition metal ion detection.

## Data Availability

The datasets and materials used and/or analyzed during the current study are available from the corresponding authors upon reasonable request.

## Abbreviations

CHMPMBS	2-[(carbamothioylhydrazono)methyl-phenyl-4-methylbenzenesulfonate
WHO	World Health Organization
ESI-MS	Electrospray Ionization–Mass Spectrometry
µM	Micromolar
THF	Tetrahydrofuran
PBS	Phosphate-Buffered Saline
DMSO	Dimethyl Sulfoxide
DMFO	Dimethylformamide
EDTA	Ethylenediaminetetraacetic acid
LOD	Limit of detection
DBQA	N-(4-(dimethylamino) benzylidene) quinolone-3-amine
HEPES	N-(2-Hydroxyethyl)piperazine-N′-2-ethanesulfonic acid
